# Accelerated cardiac T_1_ mapping in four heartbeats with inline MyoMapNet: a deep learning-based T_1_ estimation approach

**DOI:** 10.1186/s12968-021-00834-0

**Published:** 2022-01-06

**Authors:** Rui Guo, Hossam El-Rewaidy, Salah Assana, Xiaoying Cai, Amine Amyar, Kelvin Chow, Xiaoming Bi, Tuyen Yankama, Julia Cirillo, Patrick Pierce, Beth Goddu, Long Ngo, Reza Nezafat

**Affiliations:** 1 Department of Medicine (Cardiovascular Division), Beth Israel Deaconess Medical Center and Harvard Medical School, 330 Brookline Avenue, MA 02215 Boston, USA; 2 Siemens Medical Solutions USA, Inc, Boston, MA USA; 3 Siemens Medical Solutions USA, Inc, Chicago, IL USA

**Keywords:** Inversion-recovery cardiac T_1_ mapping, Machine learning, Myocardial tissue characterization, Cardiovascular magnetic resonance

## Abstract

**Purpose:**

To develop and evaluate MyoMapNet, a rapid myocardial T_1_ mapping approach that uses fully connected neural networks (FCNN) to estimate T_1_ values from four T_1_-weighted images collected after a single inversion pulse in four heartbeats (Look-Locker, LL4).

**Method:**

We implemented an FCNN for MyoMapNet to estimate T_1_ values from a reduced number of T_1_-weighted images and corresponding inversion-recovery times. We studied MyoMapNet performance when trained using native, post-contrast T_1_, or a combination of both. We also explored the effects of number of T_1_-weighted images (four and five) for native T_1_. After rigorous training using *in-vivo* modified Look-Locker inversion recovery (MOLLI) T_1_ mapping data of 607 patients, MyoMapNet performance was evaluated using MOLLI T_1_ data from 61 patients by discarding the additional T_1_-weighted images. Subsequently, we implemented a prototype MyoMapNet and LL4 on a 3 T scanner. LL4 was used to collect T_1_ mapping data in 27 subjects with inline T_1_ map reconstruction by MyoMapNet. The resulting T_1_ values were compared to MOLLI.

**Results:**

MyoMapNet trained using a combination of native and post-contrast T_1_-weighted images had excellent native and post-contrast T_1_ accuracy compared to MOLLI. The FCNN model using four T_1_-weighted images yields similar performance compared to five T_1_-weighted images, suggesting that four T_1_ weighted images may be sufficient. The inline implementation of LL4 and MyoMapNet enables successful acquisition and reconstruction of T_1_ maps on the scanner. Native and post-contrast myocardium T_1_ by MOLLI and MyoMapNet was 1170 ± 55 ms vs. 1183 ± 57 ms (P = 0.03), and 645 ± 26 ms vs. 630 ± 30 ms (P = 0.60), and native and post-contrast blood T_1_ was 1820 ± 29 ms vs. 1854 ± 34 ms (P = 0.14), and 508 ± 9 ms vs. 514 ± 15 ms (P = 0.02), respectively.

**Conclusion:**

A FCNN, trained using MOLLI data, can estimate T_1_ values from only four T_1_-weighted images. MyoMapNet enables myocardial T_1_ mapping in four heartbeats with similar accuracy as MOLLI with inline map reconstruction.

**Supplementary Information:**

The online version contains supplementary material available at 10.1186/s12968-021-00834-0.

## Introduction

Cardiovascular magnetic resonance (CMR) myocardial T_1_ and extracellular volume (ECV) mapping enable non-invasive quantification of diffuse interstitial fibrosis [1]. Generally, myocardial T_1_ mapping consists of a preparation pulse and collection of a series of images to sample the recovering longitudinal magnetization at different time points. Based on the evolution of the longitudinal magnetization across the acquired T_1_-weighted images, T_1_ at each pixel could be determined [2–4]. Over the past decade, there have been significant advances in myocardial T_1_ mapping sequence with different choices of magnetization preparation (e.g., inversion [5, 6], saturation [3, 7], or a combination of both [8]), number of collected T_1_-weighted images, and recovery period between different imaging blocks [6, 9]. Trade-offs depend on accuracy and precision [2, 10]. There are also differences in terms of coverage (e.g., single 2D, interleaved multislice 2D, or 3D) and respiratory motion compensation (free breathing vs. breath-holding) [11–15]. There is also growing interest in using a single sequence to simultaneously measure different tissue relaxation times [16–22]. These approaches often require a more complicated fitting model with more parameters, resulting in a loss of precision and significantly longer reconstruction time, which reduce their clinical utility.

Among different myocardial T_1_ mapping sequences, Modified Look-Locker inversion recovery (MOLLI) is the most widely used due to its high precision and broad vendor availability [5]. Within a single breath-hold scan, MOLLI performs three sets of Look-Locker inversion-recovery experiments to collect 3, 3, and 5 electrocardiogram (ECG)-triggered T_1_-weighted images, respectively, with 3 resting heartbeats between every two Look-Locker experiments for magnetization recovery. This acquisition scheme is referred to as MOLLI3(3)3(3)5. A 3-parameter inversion-recovery model with Look-Locker correction is used to calculate T_1_. However, MOLLI3(3)3(3)5 suffers from inaccurate T_1_ estimates and long 17 heartbeat breath-holding time. Subsequently, several derivations of MOLLI have been proposed to improve accuracy, precision, or shorten imaging time. For example, MOLLI5(3)3 and MOLLI4(1)3(1)2 protocols both reduce single breathholding to 11 heart beats(9), and the latter improves precision for short T_1_ times. Shortened MOLLI (ShMOLLI) uses a 5(1)1(1)1 scheme to further reduce imaging time and alleviate effects of heart rate variation by using a conditional fitting algorithm [6]. Inversion group fitting has also been proposed, consisting of a shorter waiting period between Look-Locker experiments, albeit with lower precision [23, 24].

Alternatives to standard curve-fitting techniques in parametric mapping include dictionary-based reconstruction [16, 25, 26], simulated signal recovery [27], and machine learning [28, 29]. Shao et al. used Bloch equation simulation with slice profile correction to model the signal evolution for MOLLI T_1_ accuracy [27]. They extended this algorithm using deep learning (DL) for rapid T_1_ map reconstruction [30]. Similarly, Zhang et al. and Hamilton et al. used DL to rapidly reconstruct T_1_ and T_2_ maps from images collected using MR fingerprinting [29, 31]. To reduce motion artifacts, an interleaved T_1_ mapping sequence with radial sampling used a convolutional neural network model to reconstruct highly accelerated T_1_-weighted image to minimize the acquisition window of the single-shot image [32]. DL was also recently used for joint saturation- and inversion-recovery T_1_ mapping to improve precision [33]. These studies indicated that DL has the potential to improve myocardial tissue characterization by increasing precision, reducing reconstruction time, decreasing motion sensitivity, and addressing imaging confounders of the myocardial T_1_ mapping sequence. However, none reduce the overall scan time for myocardial T_1_ mapping.

In this study, we sought to develop and evaluate a rapid myocardial T_1_ mapping technique, referred to as *MyoMapNet*, to perform myocardial T_1_ mapping in 4 heartbeats with similar accuracy and precision as conventional MOLLI. A single Look-Locker experiment is performed to collect four T_1_-weighted images (LL4), which are subsequently used in a fully connected neural network (FCNN) to rapidly build T_1_ map. We hypothesize that a DL-based method can learn T_1_ from a limited number of T_1_-weighted samples along the inversion-recovery curve. After initial development and evaluation, we implemented a MyoMapNet prototype on the scanner for seamless integration into the T_1_ mapping acquisition and reconstruction clinical workflow.

## Methods

### MyoMapNet

**Fig. 1:**
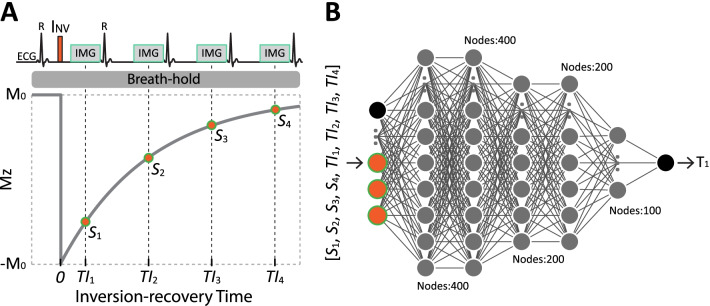
Image acquisition and map estimation in MyoMapNet. **A** The proposed breath-hold T_1_ mapping sequence (referred to as Look-Locker 4 (LL4)) consists of an inversion pulse (INV) followed by four single-shot Electrocardiogram (ECG)-triggered images. **B** A fully connected neural network (FCNN) is then used to determine T_1_ at each pixel with T_1_-weighted signals (i.e., S_i_) and inversion times (i.e., TI_i_)

LL4 performs an inversion pulse followed by four ECG-triggered single-shot balanced steady-state free precession (bSSFP) images acquired on successive cardiac cycles within a single breath-hold (Fig. 1A). The inversion-recovery time, defined as the period between the inversion pulse and the acquisition of the central k-space line, is TI_1_ for the first image and TI_1_ + (*n*-1)*RR for the image acquired in the *n*th cardiac cycle. Subsequently, an FCNN is used to estimate T_1_ from four T_1_-weighted signals with corresponding TIs at each pixel (Fig. 1B). A detailed description of the FCNN architecture and optimization are presented in the training section.

We first sought to investigate the performance of MyoMapNet trained with three different datasets: (1) using only the first 4 images from native T_1_ mapping data by MOLLI5(3)3 (MyoMapNet^4, PreGd^); (2) using only the first 4 images from post-contrast T_1_ mapping data by MOLLI4(1)3(1)2 (MyoMapNet^4, PostGd^); and (3) using the first 4 images of both native and post-contrast T_1_ mapping data according to their respective MOLLI protocols (MyoMapNet^4, Pre+PostGd^).

Considering the potential loss of T_1_ precision using only four T_1_-weighted images, we also investigated the model's performance using five T_1_-weighted images. Given that existing MOLLI data were used for training, only MOLLI5(3)3 acquired prior to contrast injection was available. We therefore only evaluated MyoMapNet with five native T_1_-weighted signals. We refer to this model as MyoMapNet^5, PreGd^. Additional file 1: Table S1 summarizes inputs and nature of data used for training of each model. We subsequently used these four models to estimate phantom and in-vivo T_1_ with and without contrast to determine whether *two separate* FC networks for native and post-contrast T_1_ estimation were needed or if a *single* model for a *single sequence* could be used to simplify imaging and map estimation.

### Existing data for training, validation, and testing

**Fig. 2:**
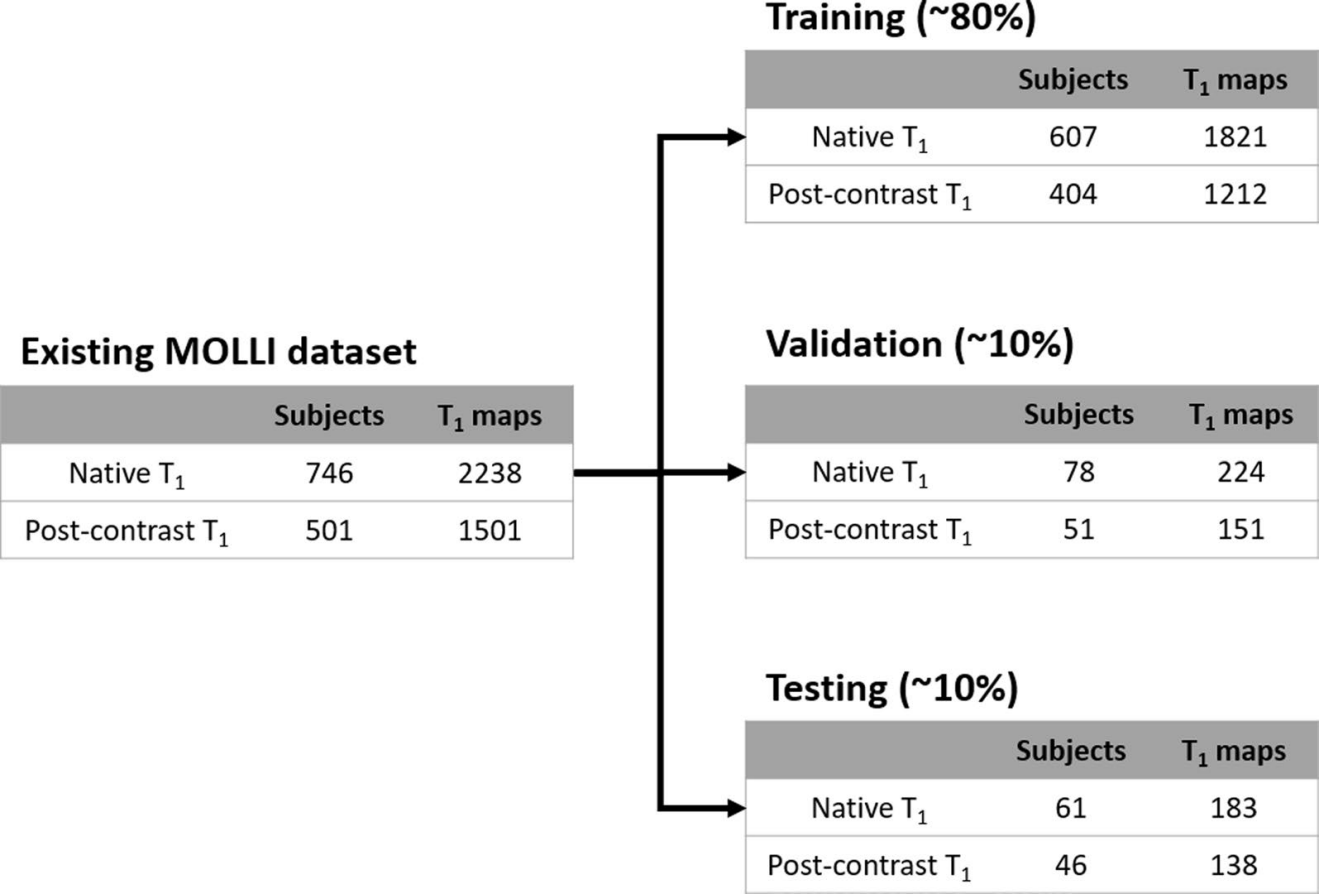
Summary of existing MOLLI T_1_ mapping data used for training, validation, and testing, collected in patients undergoing a clinical CMR exam. Native and post-contrast T_1_ mapping were performed using MOLLI5(3)3 and MOLLI4(1)3(1)2, respectively

T_1_ mapping data from 749 patients (407 male; 16–96 yrs) undergoing MOLLI scans between Jan 1, 2019, and Oct 15, 2020, were retrospectively collected (Fig. 2). Patients were referred for a clinical CMR exam for various cardiovascular indications. Our local institutional review board approved use of in-vivo data for research with a consent waiver. Patient information was handled in compliance with the Health Insurance Portability and Accountability Act (HIPAA).

Every patient had either native or both native and post-contrast T_1_-weighted images acquired by MOLLI5(3)3 and MOLLI4(1)3(1)2 for three left-ventricle (LV) short-axis view slices. All images were collected on a 3 T CMR scanner (MAGNETOM Vida, Siemens Healthineers, Erlangen, Germany) using body and spine phased-array coils. Imaging parameters used in both sequences are summarized in Additional file 1: Table S2. Post-contrast T_1_ mapping was scanned 15–20 min after injection of 0.1 mmol/kg Gd-DTPA (Gadavist, Bayer Healthcare, Berlin, Germany). The motion-correction algorithm of the vendor was used to align the myocardium across T_1_-weighted images for each scan. T_1_ maps for both sequences were calculated offline using a 3-parameter inversion-recovery model with Look-Locker correction. We randomly divided this dataset into training (~ 80%), validation (~ 10%), and testing (~ 10%) (Fig. 2).

### MyoMapNet training

MyoMapNet was implemented in Python using the PyTorch library (1.4.0). Training, validation, and testing were performed on a DGX-1 workstation (NVIDIA Santa Clara, California, USA) equipped with 88 Intel Xeon central processing units (2.20 GHz), one NVIDIA Tesla V100 graphics processing unit (GPU) with 32 GB memory and 5120 Tensor cores, and 504 GB RAM.

In the training step, we first sought to investigate the choice of hyperparameters and training performance to obtain the best model. We investigated various hyperparameters, including the number of hidden layers from 2 to 6; the number of neurons in each hidden layer (50, 100, 200, 400); activation functions such as rectified linear activation (Relu) and Leaky Relu; different sizes of mini-batches (32, 40, 64, 80); different optimizers(Adam and stochastic gradient descent (SGD)); and different learning rates (0.001, 0.01).

The model parameters consisted of all weights and biases that were learned during training by minimizing the mean absolute error (MAE):

1 $${\text{MAE}} = \frac{1}{{{\text{Number}}\,{\text{of}}\,{\text{Pixels}}}}\left| {{\text{MOLLI T}}_{1} - {\text{Myo}}\,{\text{MapNet}}\,{\text{T}}_{1} } \right|$$

To avoid overfitting or underfitting, T_1_ estimation errors of the training and validation datasets were monitored during training. For the training dataset, T_1_ estimation error over the entire image and expected T_1_ ranges (i.e., myocardium and blood) were calculated. In addition to reporting global T_1_ estimation error over the image in the validation dataset, we also monitored and reported errors for the myocardium and blood.

The trained network and instructions on how to use the network are publicly available (https://github.com/HMS-CardiacMR/MyoMapNet).

### Inline integration

**Fig. 3:**
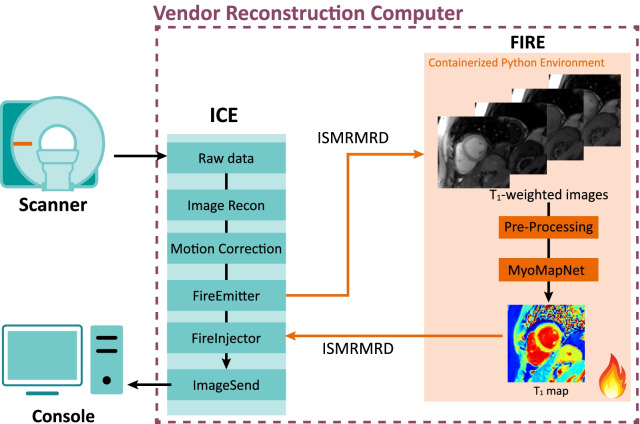
Schematic of the implemented inline integration of MyoMapNet using the Siemens Framework for Image Reconstruction (FIRE) prototype. The pre-trained MyoMapNet model was deployed in a containerized (chroot) Python 3.6 environment compatible with the FIRE framework. Data acquired on the scanner underwent standard image reconstruction and motion correction in the Siemens ICE pipeline, and T_1_-weighted images were converted into ISMRM Raw Data format (ISMRMRD) and sent to the MyoMapNet model. In the pre-processing step, the T_1_-weighted signals were normalized to the range of 0–1.1. After prediction, T_1_ map was sent back to the ICE pipeline in the ISMRMRD format where distortion correction and DICOM images were generated and displayed on the CMR console

The trained MyoMapNet prototype was deployed on a 3 T CMR scanner (MAGNETOM Vida, Siemens Healthineers, Erlangen, Germany) for inline T_1_ map building (Fig. 3). The inline integration was implemented using the Siemens Framework for Image Reconstruction (FIRE) prototype framework. Briefly, the FIRE framework provides an interface for raw data or image between the Siemens Image Reconstruction Environment (ICE) pipeline and an external environment similar to Python. The pre-trained MyoMapNet^4, Pre+PostGd^ model was deployed in a containerized (chroot) Python 3.6 environment compatible with the FIRE framework. Data acquired on the scanner underwent standard image reconstruction and motion correction in the Siemens ICE pipeline. Motion-corrected T_1_-weighted images were then converted into International Society of Magnetic Resonance in Medicine Raw Data format (ISMRMRD) [34]. Before feeding into MyoMapNet, the T_1_-weighted signals were normalized to 0–1.1. T_1_ map was then reconstructed by MyoMapNet and sent back to the ICE pipeline in ISMRMRD format, where distortion correction and DICOM images were generated and displayed on the CMR console.

### MyoMapNet performance

#### Phantom evaluation

A T1MES prototype phantom containing 12 vials with different T_1_ and T_2_ values for cardiac T_1_ mapping at 3 T was used [35]. Reference T_1_ and T_2_ of phantom vials were measured by inversion-recovery spin-echo (IR-SE) and Carr-Purcell-Meiboom-Gill spin-echo (CPMG-SE), respectively. MOLLI5(3)3, MOLLI4(1)3(1)2, and LL4 were performed at a simulated heart rate of 60 bpm. Each sequence was repeated ten times, and repetitions of all sequences were performed in random order. Imaging parameters for all sequences are described in Additional file 1: Table S2. T_1_ maps for two MOLLI sequences were fitted offline using a three-parameter inversion-recovery signal model with Look-Locker correction.

#### In-vivo evaluation using existing MOLLI data

We evaluated four trained MyoMapNet models (Additional file 1: Table S1) using existing MOLLI data. Similar to the training steps, we extracted the first four or five T_1_-weighted images of MOLLI5(3)3 or MOLLI4(1)3(1)2 and their TIs, which were then fed into MyoMapNet to predict T_1_ values. For this evaluation, we included data from 61 patients, of which 40 had both native and post-contrast T_1_ images, and 21 had only native T_1_ data. Since these datasets were not used in either training or validation, they were new to the model.

#### In-vivo evaluation using prospectively collected LL4

To further evaluate MyoMapNet performance for accelerated T_1_ mapping LL4, we prospectively recruited 28 subjects consisting of 20 patients (12 male; 61 ± 12 yrs) referred for a clinical CMR and 8 healthy subjects (5 male; 27 ± 14 yrs). These *in-vivo* experiments were HIPAA compliant and approved by our Institutional Review Board. Written informed consent was obtained from each subject prior to imaging. Native T_1_ data was collected in 25 subjects and post-contrast T_1_ data in 16 subjects. Due to IRB restrictions, gadolinium was not administered to any healthy subjects, and four patients did not receive contrast as part of their clinical protocol. In addition to clinical T_1_ mapping by MOLLI, we collected T_1_-weighted images for a single mid-LV slice using LL4 within a single breath-hold. All imaging parameters, RF shape, gradient waveforms, and timing of LL4 were identical to conventional MOLLI, with the only difference being the number of T_1_-weighted images. Imaging parameters are described in Additional file 1: Table S2. A T_1_ map using our prototype inline MyoMapNet^4, Pre+PostGd^ was reconstructed on the scanner. To further evaluate model performance, we exported images and then used each model to predict T_1_.

### Statistical analysis

For phantom T_1_, a circular region of interest (ROI) composed of ~ 120 pixels was drawn on each vial. The mean, standard deviation (SD), and coefficient of variation (CV) of T_1_ pixels within each ROI were calculated. For each sequence, mean, SD, and CV for each vial were averaged across all ten repetitions.

For each *in-vivo* T_1_ map, contours for the endo- and epicardium boundaries and blood pool were manually drawn to measure the entire LV myocardium and blood T_1_. T_1_ was reported as mean ± SD. CV was then calculated to compare the intrasubject variation. ECV was calculated for the subject who had both native and post-contrast T_1_ with their blood hematocrit sampled prior to CMR imaging. For MyoMapNet^5, PreGd^ (if any) and MyoMapNet^4, PreGd^, ECV was calculated with post-contrast T_1_ from MyoMapNet^4, PostGd^.

Bland–Altman analysis was performed to determine agreement in T_1_ or ECV values between the two methods (i.e., MOLLI and MyoMapNet). Paired Student’s t-test was also used for pair-wise comparisons. A P-value less than 0.05 was considered statistically significant. Statistical analyses were performed using GraphPad Prism (version 9.2.0, GraphPad Software, San Diego, California, USA).

## Results

### MyoMapNet training

Table 1 lists the MAE for various hyperparameters. Based on these preliminary optimization results, we chose the model with six layers. The number of neurons for each layer is 400, 400, 100, 100, 50, and 50. The activation function is Leaky Relu with a mini-batch of 64. The Adam optimizer was used with a learning rate of 0.01 and a weight decay of 0.0001.

**Table 1.** Results of hyperparameter optimization

Layers	Number of neurons in each layer	Activation function	Batch size	Learning rate	Mean Error of estimated T_1_ (ms)		
					All pixels	Myocardium	Blood
3	400, 400, 1	Leaky Relu	64	0.01	145.5	-26.7	17.9
4	400, 200, 100, 1	Leaky Relu	32	0.01	145.8	-22.6	9.1
5	400, 400, 200, 100, 1	Relu	64	0.01	176.5	26.2	192.8
**6**	**400, 400, 200, 200, 100, 1**	**Leaky Relu**	**64**	**0.01**	**111.8**	**-9.4**	**-7.9**
7	400, 400, 400, 400, 200, 100, 1	Relu	64	0.001	137.6	18.1	60.2

Adam optimizer yields the best result in all experiments. The selected hyperparameters for MyoMapNet are highlighted as bold

**Fig. 4:**
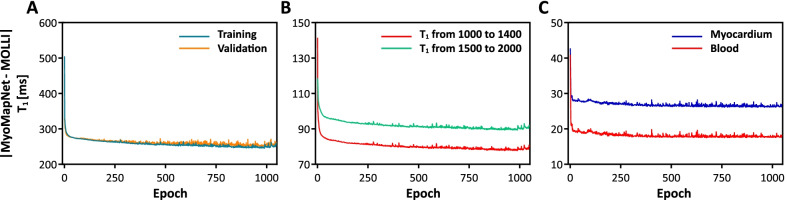
Loss curves for MyoMapNet^4, Pre+PostGd^ calculated across the entire image for both training and validation (**A**), for specific ranges of T_1_ using training dataset (**B**), and for the myocardium and blood pool of the validation dataset (**C**). Losses for both training and validation decrease with each epoch until a point of stability at ~ 1000 epochs, when learning is stopped by an early stopping, as 70 epochs were passed without improvement

Loss curves for MyoMapNet^4, Pre+PostGd^, calculated over the entire image, show the stability of the model (Fig. 4A). The training and validation losses decrease to the point of stability with small differences between validation and training. For the training dataset, the loss curves for T_1_ ranged from 1000 to 1400 ms and from 1500 to 2000 ms demonstrates similar performance as the loss calculated over the entire image (Fig. 4B). A similar observation was made when calculating the losses in the validation dataset (Fig. 4C). For the validation dataset, MAE for myocardium and blood were ~ 27 ms and ~ 10 mm, respectively.

### Phantom evaluation

**Fig. 5:**
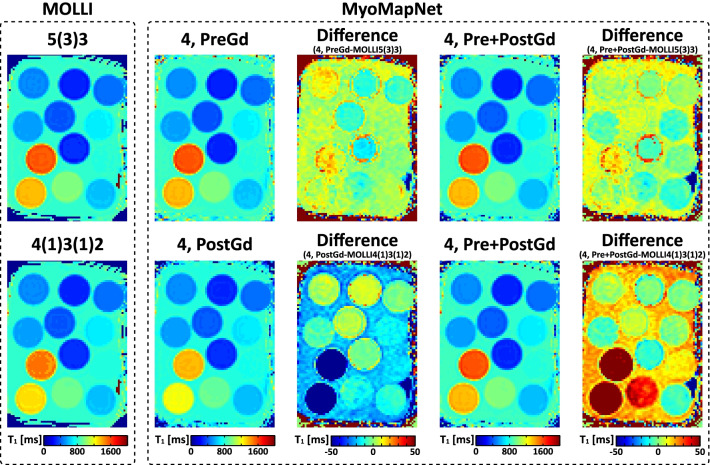
Phantom T_1_ maps from two MOLLI sequences (MOLLI5(3)3 and MOLLI4(1)3(1)2) and LL4 with different MyoMapNet models. T_1_ difference maps between them were included. All MyoMapNet models show similar map quality, except for the model trained using only post-contrast T_1_ mapping data. In the T_1_ analysis for the post-contrast models, vials with larger T_1_ values (> 900 ms) were excluded. While the model trained using only *in-vivo* data, phantom data show that the model can reliably estimate T_1_ values for vials with T_1_/T_2_s that are not necessarily well represented in the training dataset

**Fig. 6:**
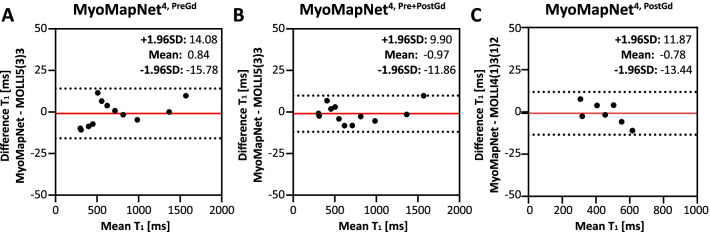
Bland–Altman plots for examining the phantom T_1_ agreement between MyoMapNet and MOLLI. The red line indicates the mean difference, and the dotted lines show the 95% confidence interval on the limits of agreement. For post-contrast T_1_ evaluation (MyoMapNet^4, PostGd^ and MOLLI4(1)3(1)2), phantom vials with T_1_ larger than 900 ms were excluded

There was no significant visual difference between phantom T_1_ maps scanned by MOLLI and LL4 with three MyoMapNet models (Fig. 5). Bland–Altman analysis (Fig. 6) shows excellent agreement between MyoMapNet and MOLLI with negligible bias in T_1_ estimate (mean bias of less than 1 ms). There was no difference in SD or CV between MOLLI and MyoMapNet, indicating the similar precision among different methods (Additional file 1: Table S3).

### In-vivo evaluation using existing data

**Fig. 7:**
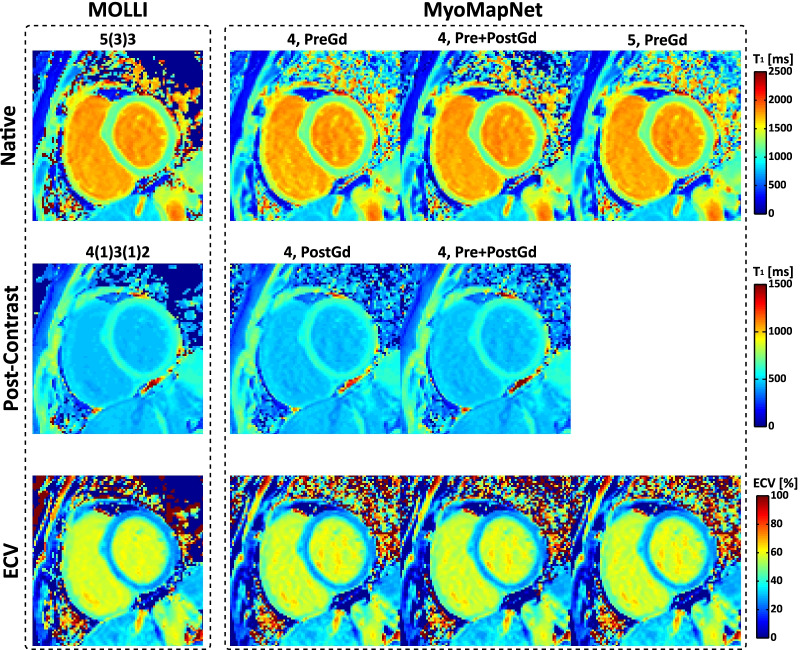
Representative native, post-contrast T_1_, and ECV maps by MOLLI and MyoMapNet from a subject in the existing MOLLI dataset. For MyoMapNet images, T_1_-weighted images from MOLLI were extracted; therefore, both MOLLI and MyoMapNet were reconstructed from the same scan. For MyoMapNet ^4, PreGd^ and MyoMapNet ^5, PreGd^, the ECV map was reconstructed with post-contrast T_1_ map from MyoMapNet ^4, PostGd^

**Fig. 8:**
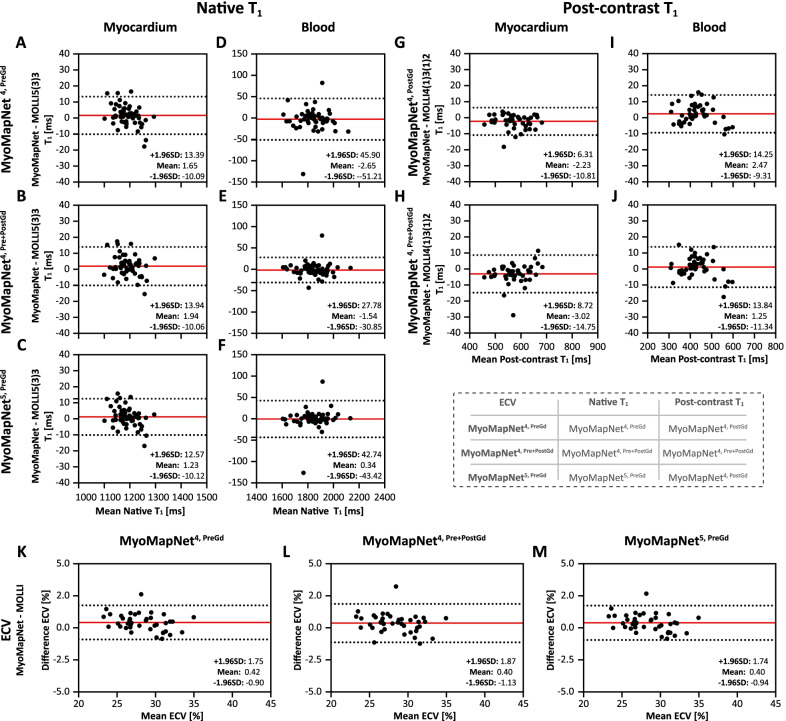
Bland–Altman plots showing individual patient comparisons between MyoMapNet and two MOLLI sequences for myocardium and blood T_1_ in the existing MOLLI data. Mean difference and 95% limits of agreement are indicated as red and dotted lines, respectively. Each data point was averaged across three left ventricular slices of one patient. Native and post-contrast T_1_ used for calculating ECV are indicated for each method

T_1_ maps for all subjects across all methods are available on our laboratory Harvard Dataverse. Visually, both maps from MOLLI and MyoMapNet have good image quality with homogeneous signal across the whole LV myocardium and clear boundaries (Fig. 7). Mean, SD, and CV values of native/post-contrast T_1_ and corresponding ECV values (if any) averaged across all subjects across all methods are summarized in Table 2 and Additional file 1: Table S4. For both native myocardium and blood T_1_, excellent agreement was achieved between MyoMapNet and MOLLI5(3)3 with a mean T_1_ difference of 2 ms and -3 ms (MyoMapNet ^4, PreGd^ vs. MOLLI5(3)3), 2 ms and -2 ms (MyoMapNet ^4, Pre+PostGd^ vs. MOLLI5(3)3), and 1 ms and 0 ms (MyoMapNet ^5, PreGd^ vs. MOLLI5(3)3). The 95% confidence interval (CI) for T_1_ differences between MyoMapNet and MOLLI5(3)3 ranged from -10 ms to 14 ms for myocardium and ranged from -51 ms to 46 ms for blood. Bland–Altman analysis also showed excellent agreement between MyoMapNet and MOLLI4(1)3(1)2 for post-contrast T_1_ estimation (Fig. 8). The mean myocardium and blood T_1_ difference between them was -2 ms and 2 ms (MyoMapNet ^4, PostGd^ vs. MOLLI4(1)3(1)2), and -3 ms and 1 ms (MyoMapNet ^4, Pre+PostGd^ vs. MOLLI4(1)3(1)2), respectively. The corresponding 95% CI for T_1_ difference ranged from -15 ms to 9 ms for myocardium and from -11 ms to 12 ms for blood. The mean difference in ECV between MyoMapNet and MOLLI was ~ 0.4% with 95% CI from -1.1% to 1.9% (all P < 0.05) (Fig. 8).

**Table 2.** Native, post-contrast T_1_ and corresponding ECV for the esixting data by MyoMapNet and MOLLI. MyoMapNet T_1_ were calculated by discarding T_1_-weighted images from the MOLLI sequence

	Myocardium	Blood
Native T_1_ (ms)		
MyoMapNet^4, Pre^	1190 ± 38^§^	1847 ± 99
MyoMapNet^4, Pre+PostGd^	1190 ± 39^§^	1849 ± 100
MyoMapNet^5, Pre^	1190 ± 38	1850 ± 104
MOLLI5(3)3	1188 ± 40	1850 ± 100
Post-Contrast T_1_ (ms)		
MyoMapNet^4, PostGd^	573 ± 54^§^	432 ± 69^§^
MyoMapNet^4, Pre+PostGd^	572 ± 56^§^	430 ± 68
MOLLI4(1)3(1)2	575 ± 54	429 ± 70
ECV (%)		
MyoMapNet^4, Pre^	28.5 ± 2.7^§^	
MyoMapNet^4, Pre+PostGd^	28.5 ± 2.8^§^	
MyoMapNet^5, Pre^	28.5 ± 2.7^§^	
MOLLI	28.1 ± 2.9	

*ECV* Extracellular volume. Mean and standard deviation were calculated by averaging the corresponding results of each subject across all subjects

^§^p-value < 0.05 when compared to MOLLI5(3)3 or MOLLI4(1)3(1)2

In terms of precision (Additional file 1: Table S4**)**, SD of the myocardium and blood T_1_ from MyoMapNet were ~ 2 ms and ~ 4 ms slightly higher than those from MOLLI5(3)3, and ~ 5 ms and ~ 7 ms higher than those from MOLLI4(1)3(1)2 (all P < 0.05), respectively. The native myocardium and blood T_1_ CV were 5.0% and 2.0% by MOLLI or all three MyoMapNet models (all P < 0.05). For post-contrast myocardium and blood T_1_, CV was 5.2% and 2.2% from MOLLI4(1)3(1)2, and ~ 6.0% and 3.7% from MyoMapNet models (all P < 0.05), respectively.

We found that MyoMapNet^5, PreGd^ with 5 T_1_-weighted images did not significantly improve T_1_ precision compared to MyoMapNet^4, PreGd^ or MyoMapNet^4, Pre+PostGd^ with only 4 T_1_-weighted images for native T_1_ or both native and post-contrast T_1_. The latter two could again save ~ 1 s imaging time. Therefore, MyoMapNet^5, PreGd^ was no longer used in any prospective evaluations.

### In-vivo evaluation using LL4 data

**Fig. 9:**
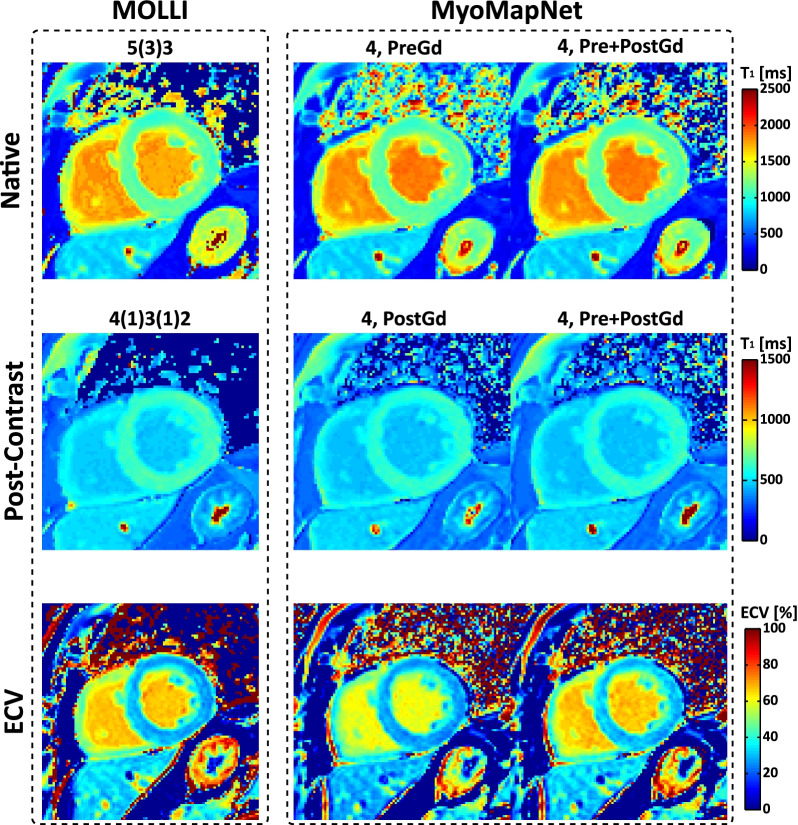
Native, post-contrast T_1_, and ECV maps by LL4 with MyoMapNet and MOLLI. In this case, maps are calculated from two different scans, one from conventional MOLLI and one from LL4

**Fig. 10:**
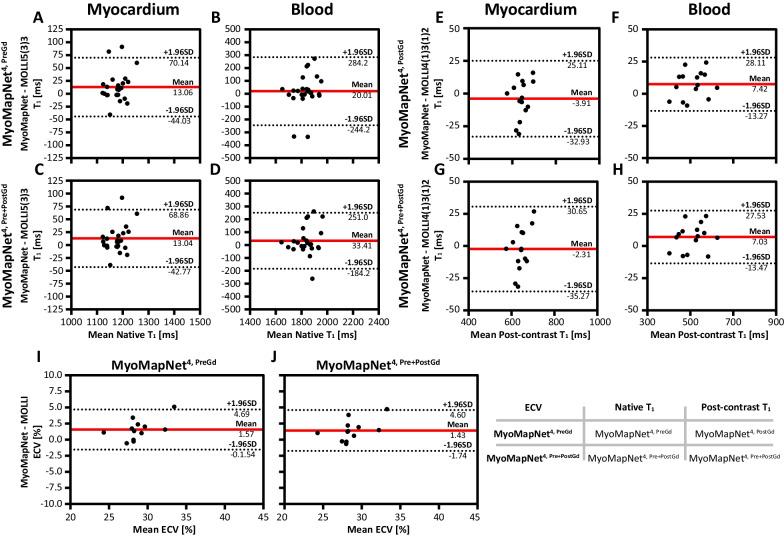
Bland–Altman plots comparing myocardium and blood T_1_ and extracellular volume fraction (ECV) values between LL4 with MyoMapNet and MOLLI, acquired in two separate scans. Mean difference and 95% limits of agreement are indicated as red and dotted lines, respectively. Each data point represents one patient. Native and post-contrast T_1_ used for ECV measurement are indicated for each method

In-vivo scanning by LL4 was successfully completed in all subjects. In maps from all MyoMapNet models and two MOLLI sequences, myocardium and blood had homogeneous signals (Fig. 9). Mean T_1_ differences in native myocardium and blood between MyoMapNet and MOLLI was 13 ms and 20 ms (MyoMapNet^4, PreGd^ vs. MOLLI5(3)3), and 13 ms and 33 ms (MyoMapNet^4, Pre+PostGd^ vs. MOLLI5(3)3) (Fig. 10A–D). Mean post-contrast myocardium and blood T_1_ difference was -4 ms and 7 ms between MyoMapNet^4, PostGd^ and MOLLI4(1)3(1)2, and -2 ms and 7 ms between MyoMapNet^4, Pre+PostGd^ and MOLLI4(1)3(1)2, respectively (Fig. 10E–H). ECV for MOLLI and MyoMapNet were ~ 28% and ~ 30% (All P < 0.05, Fig. 10I–J and Table 3).

**Table 3.** Native and post-contrast T_1_ values for prospectively collected data by LL4 with MyoMapNet and MOLLI

	Myocardium	Blood
Native T_1_ (ms)		
MyoMapNet^4, Pre^	1183 ± 56^§^	1840 ± 34
MyoMapNet^4, Pre+PostGd^	1183 ± 57^§^	1854 ± 34
MOLLI5(3)3	1170 ± 55	1820 ± 29
Post-Contrast T_1_ (ms)		
MyoMapNet^4, PostGd^	641 ± 29	515 ± 15^§^
MyoMapNet^4, Pre+PostGd^	630 ± 30	514 ± 15^§^
MOLLI4(1)3(1)2	645 ± 26	508 ± 9

^§^p-value < 0.05 when compared to MOLLI5(3)3 or MOLLI4(1)3(1)2

SD for native myocardium T_1_ did not differ between MOLLI5(3)3 and MyoMapNet^4, PreGd^ (P = 0.95) or MyoMapNet^4, Pre+PostGd^ (P = 0.59) (Additional file 1: Table S5). SD of the native blood T_1_ from MyoMapNet was higher than that from MOLLI5(3)3 (34 ms vs. 29 ms, all P < 0.05). For post-contrast T_1_, SD of myocardium T_1_ was 26 ms from MOLLI4(1)3(1)2 and ~ 30 ms from MyoMapNet (MOLLI4(1)3(1)2 vs. MyoMapNet^4, PostGd^: P = 0.24; MOLLI4(1)3(1)2 vs. MyoMapNet^4, Pre+PostGd^: P = 0.04). Similar to the native T_1_ measurement, the SD for blood T_1_ from MyoMapNet was higher than that from MOLLI4(1)3(1)2 (15 ms vs. 9 ms, all P < 0,05). The mean CV of native myocardium and blood T_1_ was ~ 5% and 2% by MOLLI5(3)3 and MyoMapNet. The CV of the post-contrast T_1_ by MyoMapNet was higher than those by MOLLI5(3)3 (myocardium: 4.3% vs. 3.8%; blood: 2.7% vs. 1.7%, all P < 0,05; Additional file 1: Table S5).

## Discussion

In this study, we developed and evaluated MyoMapNet, an accelerated myocardial T_1_ mapping approach that can perform T_1_ mapping within four heartbeats. An FCNN was trained to estimate T_1_ values using four T_1_ weighted signals sampled along a single Look-Locker inversion-recovery curve. Through phantom and *in-vivo* validation using existing data and prospectively collected data, we demonstrated that a MyoMapNet model trained with a combination of native and post-contrast T_1_ mapping data can be used to calculate T_1_ for both native and post-contrast T_1_ mapping. Thus, a single FCNN model for both native and post-contrast T_1_ mapping using a single LL4 sequence is sufficient. Our inline implementation of the model demonstrated feasibility for rapidly deploying such a model on the scanner.

It is well established that MOLLI has several confounders (e.g., heart rate, T_2_ sensitivity, magnetization transfer) [2, 36, 37]. In addition, MOLLI T_1_ is lower than actual T_1_ due to the intermittent bSSFP readout compared to a standard continuous gradient-echo Look-Locker acquisition. Since we used MOLLI data for training the model, the current implementation of MyoMapNet has theoretically similar limitations as MOLLI. Alternative approaches in which the model is trained using a more accurate T_1_ mapping sequence could improve the accuracy of MypMapNet. Numerical simulations using the Bloch equation could be used to generate synthetic data for training with the ground truth. A combination of simulated and *in-vivo* signals could also be used to train the network to further improve MyoMaoNet accuracy and robustness.

We investigated MyoMapNet performance in terms of accuracy and precision using only four T_1_-weighted images and compared it with conventional MOLLI. However, one can create T_1_ map using a reduced number of T_1_-weighted images using a conventional 2 or 3-parameter fitting model. Fitts et al. [38] proposed an arrhythmia insensitive rapid cardiac T_1_ mapping pulse sequence based on only two T_1_-weighted images; however, estimated T_1_ values differed with conventional MOLLI [39]. Our group had previously compared MyoMapNet versus a conventional fitting model using the same number of T_1_-weighted imaging. In comparison to MyoMapNet, conventional fitting had lower precision and larger bias. Additionally, curve-fitting performance differs for images with different signal-to-noise ratios and T_1_ values. For the existing MOLLI dataset used in this study, we performed a head-to-head comparison of both approaches (results are included in Supplementary Materials), which also demonstrated similar loss of precision and increased bias.

We randomly divided our existing MOLLI dataset into 80%-10%-10% for training, validation, and testing. While there is no optimal split percentage, using 60–80% of the data for training is quite common. The model performance could be impacted by the splitting ratio [40]. Since we used independent prospectively collected data for further evaluation of the final trained model and its generalizability, we did not investigate different data splitting ratios. An alternative approach would be to stratify the data based on the distribution of T_1_ values, so all expected ranges of T_1_ are represented in the training dataset. Further studies are warranted to further improve the training and generalizability of the model by studying the optimal dataset size and splitting ratio.

We used an FCNN for MyoMapNet to estimate T_1_ from the reduced number of T_1_-weighted images. The evaluation indicated that such an FCNN model had comparable precision with MOLLI and was better than curve-fitting method (Additional file 2: Figures S3 and S4). In this model, each pixel is treated independently. Alternatively, a convolutional neural network that incorporates data from neighboring pixels could also be used. In a prior preliminary study, we implemented such a model (data not shown) and observed superior noise performance that could potentially improve T_1_ precision. Further investigation is warranted to evaluate alternative DL models for MyoMapNet.

Respiratory motion can cause image artifacts in myocardial T_1_ mapping. Breath-holding or free-breathing imaging with slice tracking in combination with image registration has been used to reduce effects of motion on the parametric mapping [41–43]. In MyoMapNet, we used motion correction to remove any potential misalignment between different T_1_-weighted images [44]. Further investigation is needed to evaluate whether the breath-hold requirement can be potentially eliminated to reduce patient burden.

We collected prospectively accelerated data to further evaluate MyoMapNet beyond the existing dataset. Although both sequences use similar imaging parameters, there are differences such as breath-holding and in-flow. This was reflected in the greater difference between the two methods in the experiment with prospectively accelerated data than existing MOLLI data. However, such evaluation is necessary to evaluate the performance of an accelerated method in CMR. An inline implementation of MyoMapNet substantially facilitates prototyping and testing of different models on the scanner. We are also taking additional steps to make MyoMapNet and its inline implementation freely available.

DL is rapidly improving the clinical workflow of myocardial tissue characterization. Recent studies have demonstrated the potential of DL to automate analysis and image quality control [45–48]. These methods could automatically perform motion correction, segmentation, and parameter quantification, thereby reducing the burden of manual analysis and observer-related variability. MyoMapNet could be easily integrated with an automated analysis and quality control method to facilitate rapid data collection and the analysis workflow.

In T_1_ mapping, the choice of inversion time can impact the accuracy and precision [49]. In LL4, after the inversion pulse, samplings along the relaxation curve are separated by the cardiac cycle. Hence, the effective inversion-recovery times are determined by the RR interval length [2, 8]. Except for the first image shortly acquired after the inversion pulse, the rest of the images have a long inversion-recovery time (> RR interval length). This would reduce sensitivity to T_1_ relaxation when the patient has a low heart rate or mis-triggered heartbeat during imaging, which impacts the T_1_ map quality, especially post-contrast T_1_ [49]. In our study, we did not investigate the optimal choice of inversion time of the first T_1_-weighted images, as we used conventional MOLLI sequence timing and parameters. Other acquisition schemes can potentially be developed to reduce sensitivity to heart rate and improve performance for short T_1_ values.

## Limitations

Our study has several limitations. We did not evaluate the optimal choice of DL architecture; however, the results from the FCNN show excellent agreement with MOLLI. We used existing MOLLI data for training, and it is widely known that MOLLI has intrinsic underestimation. We did not investigate how different confounders, such as B_1_ or B_0_ inhomogeneity, could impact MyoMapNet performance. We used a large patient dataset with various clinical indications; however, we did not evaluate the performance of MyoMapNet for specific cardiomyopathies with abnormal T_1_ values. Finally, data from a single vendor and field strength were used for training, and the generalizability of the trained network should be studied.

## Conclusion

The MyoMapNet enables fast myocardial T_1_ mapping from only four T_1_-weighted images collected by a single Look-Locker sequence, leading to shorter scan time and rapid map reconstruction.

## Supplementary Information

**Additional file 1: Table S1.** Notation, input, training, prediction, and application of each model. **Table S2.** Imaging parameters for all sequences used in this study. **Table S3.** Mean, standard deviation and coefficient of variation (CV) of T_1_ of each phantom estimated by MOLLI and three MyoMapNet models. **Table S4.** Standard deviation and coefficient of variation (CV) of T_1_ of existing data estimated by MOLLI and MyoMapNet. **Table S5.** Standard deviation and coefficient of variation (CV) of T_1_ for prospectively collected data by MOLLI and LL4 with MyoMapNet. **Table S6.** Native, post-contrast T_1_, and corresponding ECV for existing MOLLI data estimated by curve-fitting methods.

**Additional file 2: Figure S1. **Simulation results. Bland–Altman plots show the mean difference and 95% limits of agreement in simulated T_1_ with different signal-to-noise (SNR) between MyoMapNet and MOLLI5(3)3, and LL5-3^P^-fitting and MOLLI5(3)3. **Figure S2.** Representative in-vivo T_1_ and corresponding ECV maps from the different number of T_1_-weighted images of MOLLI using MyoMapNet and curve-fitting methods. **Figure S3.** Comparison between MyoMapNet and curve-fitting methods (LL4- and LL5-3^P^-fitting) for myocardium T_1_ from four or five T_1_ weighted images, using MOLLI as reference: MOLLI5(3)3 for native T_1_ and MOLLI4(1)3(1)2 for post-contrast T_1_. Each data point was averaged across three LV slices for each patient. Mean difference and 95% limits of agreement are indicated as red and dotted lines, respectively. **Figure S4.** Comparison of MyoMapNet and curve-fitting methods for blood T_1_ from a few T_1_-weighted images (4 or 5) to reference sequences. MOLLI5(3)3 was the reference for native T_1_, and MOLLI4(1)3(1)2 was the reference for post-contrast T_1_. Mean difference and 95% limits of agreement are indicated as red and dotted lines for each subfigure, respectively. **Figure S5.** Comparing ECV from MyoMapNet models and curve fitting (LL4- and LL5-3^P^-fitting) to MOLLI. Mean difference and 95% limits of agreement are indicated as red and dotted lines for each subfigure, respectively. Native and post-contrast T_1_ values used for ECV measurement are indicated for each method.

## Data Availability

MyoMapNet is an investigational technique and not available by the vendor as a research tool or product. MyoMapNet codes are openly available on GitHub (https://github.com/HMS-CardiacMR/MyoMapNet). All reconstructed T_1_ maps are available on Harvard dataverse (https://dataverse.harvard.edu/dataverse/cardiacmr), reference number (https://doi.org/10.7910/DVN/5MZYAH).

## Abbreviations

2D: Two dimensional
3D: Three dimensional
bSSFP: Balanced steady-state free precession
CI: Confidence interval
CMR: Cardiovascular magnetic resonance
CPMG-SE: Carr-Purcell-Meiboom-Gill spin echo
CPU: Central processing unit
CV: Coefficient of variation
DL: Deep learning
ECG: Electrocardiogram
ECV: Extracellular volume
FCNN: Fully connected neural network
FIRE: Framework for image reconstruction environment
Gd: Gadolinium
GPU: Graphics processing units
ICE: Siemens Image reconstruction environment.
IR-SE: Inversion recovery spin echo
LL4: Look-Locker in 4 heartbeats
ISMRMRD: International Society of Magnetic Resonance in Medicine Raw Data format
LV: Left ventricle/left ventricular
MAE: Mean absolute error
MOLLI: Modified Look-Locker inversion recovery
NN: Neural networks
ROI: Region of interest
RR: Duration of one heartbeat
SD: Standard deviation
ShMOLLI: Shortened modified Look-Locker inversion recovery
SNR: Signal-to-noise ratio
TI: Inversion time

## References

[1] Messroghli DR, Moon JC, Ferreira VM, Grosse-Wortmann L, He T, Kellman P, Mascherbauer J, Nezafat R, Salerno M, Schelbert EB, Taylor AJ, Thompson R, Ugander M, van Heeswijk RB, Friedrich MG (2017). Clinical recommendations for cardiovascular magnetic resonance mapping of T1, T2, T2* and extracellular volume: a consensus statement by the Society for Cardiovascular Magnetic Resonance (SCMR) endorsed by the European Association for Cardiovascular Imaging (EACVI). J Cardiovasc Magn Reson.

[2] Kellman P, Hansen MS (2014). T1-mapping in the heart: accuracy and precision. J Cardiovasc Magn Reson.

[3] Chow K, Flewitt JA, Green JD, Pagano JJ, Friedrich MG, Thompson RB (2014). Saturation recovery single-shot acquisition (SASHA) for myocardial T(1) mapping. Magn Reson Med.

[4] Xue H, Greiser A, Zuehlsdorff S, Jolly M-P, Guehring J, Arai AE, Kellman P (2013). Phase-sensitive inversion recovery for myocardial T1 mapping with motion correction and parametric fitting. Magn Reson Med.

[5] Messroghli DR, Radjenovic A, Kozerke S, Higgins DM, Sivananthan MU, Ridgway JP (2004). Modified Look-Locker inversion recovery (MOLLI) for high-resolution T1 mapping of the heart. Magn Reson Med.

[6] Piechnik SK, Ferreira VM, Dall'Armellina E, Cochlin LE, Greiser A, Neubauer S, Robson MD. Shortened Modified Look-Locker Inversion recovery (ShMOLLI) for clinical myocardial T1-mapping at 1.5 and 3 T within a 9 heartbeat breathhold. J Cardiovasc Magn Reson 2010;12: 69. 10.1186/1532-429X-12-69 PMC3001433 21092095

[7] Higgins DM, Ridgway JP, Radjenovic A, Sivananthan UM, Smith MA (2005). T1 measurement using a short acquisition period for quantitative cardiac applications. Med Phys.

[8] Weingartner S, Akcakaya M, Basha T, Kissinger KV, Goddu B, Berg S, Manning WJ, Nezafat R (2014). Combined saturation/inversion recovery sequences for improved evaluation of scar and diffuse fibrosis in patients with arrhythmia or heart rate variability. Magn Reson Med.

[9] Kellman P, Wilson JR, Xue H, Ugander M, Arai AE (2012). Extracellular volume fraction mapping in the myocardium, part 1: evaluation of an automated method. J Cardiovasc Magn Reson.

[10] Roujol S, Weingartner S, Foppa M, Chow K, Kawaji K, Ngo LH, Kellman P, Manning WJ, Thompson RB, Nezafat R (2014). Accuracy, precision, and reproducibility of four T1 mapping sequences: a head-to-head comparison of MOLLI, ShMOLLI, SASHA, and SAPPHIRE. Radiology.

[11] Weingartner S, Roujol S, Akcakaya M, Basha TA, Nezafat R (2014). Free-breathing multislice native myocardial T1 mapping using the slice-interleaved T1 (STONE) sequence. Magn Reson Med.

[12] Guo R, Chen Z, Wang Y, Herzka DA, Luo J, Ding H (2018). Three-dimensional free breathing whole heart cardiovascular magnetic resonance T1 mapping at 3 T. J Cardiovasc Magn Reson.

[13] Guo R, Cai X, Kucukseymen S, Rodriguez J, Paskavitz A, Pierce P, Goddu B, Nezafat R (2021). Free-breathing whole-heart multi-slice myocardial T(1) mapping in 2 minutes. Magn Reson Med.

[14] Weingartner S, Akcakaya M, Roujol S, Basha T, Stehning C, Kissinger KV, Goddu B, Berg S, Manning WJ, Nezafat R (2015). Free-breathing post-contrast three-dimensional T1 mapping: Volumetric assessment of myocardial T1 values. Magn Reson Med.

[15] Weingartner S, Akcakaya M, Roujol S, Basha T, Tschabrunn C, Berg S, Anter E, Nezafat R (2015). Free-breathing combined three-dimensional phase sensitive late gadolinium enhancement and T1 mapping for myocardial tissue characterization. Magn Reson Med.

[16] Hamilton JI, Jiang Y, Chen Y, Ma D, Lo WC, Griswold M, Seiberlich N (2017). MR fingerprinting for rapid quantification of myocardial T1, T2, and proton spin density. Magn Reson Med.

[17] Christodoulou AG, Shaw JL, Nguyen C, Yang Q, Xie Y, Wang N, Li D (2018). Magnetic resonance multitasking for motion-resolved quantitative cardiovascular imaging. Nat Biomed Eng.

[18] Akcakaya M, Weingartner S, Basha TA, Roujol S, Bellm S, Nezafat R (2016). Joint myocardial T1 and T2 mapping using a combination of saturation recovery and T2 -preparation. Magn Reson Med.

[19] Santini F, Kawel-Boehm N, Greiser A, Bremerich J, Bieri O (2015). Simultaneous T1 and T2 quantification of the myocardium using cardiac balanced-SSFP inversion recovery with interleaved sampling acquisition (CABIRIA). Magn Reson Med.

[20] Guo R, Cai X, Kucukseymen S, Rodriguez J, Paskavitz A, Pierce P, Goddu B, Thompson RB, Nezafat R. Free-breathing simultaneous myocardial T1 and T2 mapping with whole left ventricle coverage. Magn Reson Med 2020. 10.1002/mrm.28506 33078443

[21] Blume U, Lockie T, Stehning C, Sinclair S, Uribe S, Razavi R, Schaeffter T (2009). Interleaved T1 and T2 relaxation time mapping for cardiac applications. J Magn Reson Imaging.

[22] Kvernby S (2013). Simultaneous three-dimensional myocardial T1 and T2 mapping in one breath hold with 3D-QALAS. J Cardiovasc Magn Reson.

[23] Sussman MS, Wintersperger BJ (2019). Modified look-locker inversion recovery (MOLLI) T1 mapping with inversion group (IG) fitting - A method for improved precision. Magn Reson Imaging.

[24] Sussman MS, Yang IY, Fok KH, Wintersperger BJ (2016). Inversion group (IG) fitting: A new T1 mapping method for modified look-locker inversion recovery (MOLLI) that allows arbitrary inversion groupings and rest periods (including no rest period). Magn Reson Med.

[25] Zhu Y, Kang J, Duan C, Nezafat M, Neisius U, Jang J, Nezafat R (2019). Integrated motion correction and dictionary learning for free-breathing myocardial T1 mapping. Magn Reson Med.

[26] Doneva M, Börnert P, Eggers H, Stehning C, Sénégas J, Mertins A (2010). Compressed sensing reconstruction for magnetic resonance parameter mapping. Magn Reson Med.

[27] Shao J, Rapacchi S, Nguyen KL, Hu P (2016). Myocardial T1 mapping at 3.0 tesla using an inversion recovery spoiled gradient echo readout and bloch equation simulation with slice profile correction (BLESSPC) T1 estimation algorithm. J Magn Reson Imaging.

[28] Cohen O, Zhu B, Rosen MS (2018). MR fingerprinting Deep RecOnstruction NEtwork (DRONE). Magn Reson Med.

[29] Zhang Q, Su P, Chen Z, Liao Y, Chen S, Guo R, Qi H, Li X, Zhang X, Hu Z, Lu H, Chen H (2020). Deep learning–based MR fingerprinting ASL ReconStruction (DeepMARS). Magn Reson Med.

[30] Shao J, Ghodrati V, Nguyen KL, Hu P (2020). Fast and accurate calculation of myocardial T(1) and T(2) values using deep learning Bloch equation simulations (DeepBLESS). Magn Reson Med.

[31] Hamilton JI, Currey D, Rajagopalan S, Seiberlich N (2021). Deep learning reconstruction for cardiac magnetic resonance fingerprinting T1 and T2 mapping. Magn Reson Med.

[32] Nezafat M, El-Rewaidy H, Kucukseymen S, Hauser TH, Fahmy AS (2020). Deep convolution neural networks based artifact suppression in under-sampled radial acquisitions of myocardial T 1 mapping images. Phys Med Biol.

[33] Gatsoni O, Aletras AH, Heiberg E, Berggren K (2020). T1 Mapping By Means Of Deep Learning Neural Networks Using Both Saturation Recovery and Inversion Recovery Data.

[34] Inati SJ, Naegele JD, Zwart NR, Roopchansingh V, Lizak MJ, Hansen DC, Liu CY, Atkinson D, Kellman P, Kozerke S, Xue H, Campbell-Washburn AE, Sørensen TS, Hansen MS (2017). ISMRM Raw data format: a proposed standard for MRI raw datasets. Magn Reson Med.

[35] Captur G, Gatehouse P, Keenan KE, Heslinga FG, Bruehl R, Prothmann M, Graves MJ, Eames RJ, Torlasco C, Benedetti G, Donovan J, Ittermann B, Boubertakh R, Bathgate A, Royet C, Pang W, Nezafat R, Salerno M, Kellman P, Moon JC (2016). A medical device-grade T1 and ECV phantom for global T1 mapping quality assurance-the T1 Mapping and ECV Standardization in cardiovascular magnetic resonance (T1MES) program. J Cardiovasc Magn Reson.

[36] Chow K, Flewitt J, Pagano JJ, Green JD, Friedrich MG, Thompson RB (2012). T2-dependent errors in MOLLI T1 values: simulations, phantoms, and in-vivo studies. J Cardiovasc Magn Reson.

[37] Robson MD, Piechnik SK, Tunnicliffe EM, Neubauer S (2013). T1 measurements in the human myocardium: the effects of magnetization transfer on the SASHA and MOLLI sequences. Magn Reson Med.

[38] Fitts M, Breton E, Kholmovski EG, Dosdall DJ, Vijayakumar S, Hong KP, Ranjan R, Marrouche NF, Axel L, Kim D (2013). Arrhythmia insensitive rapid cardiac T1 mapping pulse sequence. Magn Reson Med.

[39] Hong K, Kim D (2014). MOLLI and AIR T1 mapping pulse sequences yield different myocardial T1 and ECV measurements. NMR Biomed.

[40] Xu Y, Goodacre R (2018). On splitting training and validation set: a comparative study of cross-validation, bootstrap and systematic sampling for estimating the generalization performance of supervised learning. J Anal Test.

[41] Roujol S, Foppa M, Weingartner S, Manning WJ, Nezafat R (2015). Adaptive registration of varying contrast-weighted images for improved tissue characterization (ARCTIC): application to T1 mapping. Magn Reson Med.

[42] El-Rewaidy H, Nezafat M, Jang J, Nakamori S, Fahmy AS, Nezafat R (2018). Nonrigid active shape model-based registration framework for motion correction of cardiac T1 mapping. Magn Reson Med.

[43] Bush MA, Ahmad R, Jin N, Liu Y, Simonetti OP (2019). Patient specific prospective respiratory motion correction for efficient, free-breathing cardiovascular MRI. Magn Reson Med.

[44] Xue H, Shah S, Greiser A, Guetter C, Littmann A, Jolly MP, Arai AE, Zuehlsdorff S, Guehring J, Kellman P (2012). Motion correction for myocardial T1 mapping using image registration with synthetic image estimation. Magn Reson Med.

[45] Fahmy AS, El-Rewaidy H, Nezafat M, Nakamori S, Nezafat R (2019). Automated analysis of cardiovascular magnetic resonance myocardial native T1 mapping images using fully convolutional neural networks. J Cardiovasc Magn Reson.

[46] Leiner T, Rueckert D, Suinesiaputra A, Baessler B, Nezafat R, Isgum I, Young AA (2019). Machine learning in cardiovascular magnetic resonance: basic concepts and applications. J Cardiovasc Magn Reson.

[47] Zhang Q, Hann E, Werys K, Wu C, Popescu I, Lukaschuk E, Barutcu A, Ferreira VM, Piechnik SK (2020). Deep learning with attention supervision for automated motion artefact detection in quality control of cardiac T1-mapping. Artif Intell Med.

[48] Zhu Y, Fahmy AS, Duan C, Nakamori S, Nezafat R (2020). Automated myocardial T2 and extracellular volume quantification in cardiac mri using transfer learning–based myocardium segmentation. Radiology.

[49] Akcakaya M, Weingartner S, Roujol S, Nezafat R (2015). On the selection of sampling points for myocardial T1 mapping. Magn Reson Med.

