# Supplementary material for: Accelerated cardiac T1 mapping in four heartbeats with inline MyoMapNet: a deep learning-based T1 estimation approach
Source: J Cardiovasc Magn Reson. 2022 Jan 6;24:6. doi: 10.1186/s12968-021-00834-0 (PMC8734349; doi:10.1186/s12968-021-00834-0)
Supplement: Supplementary file 1 — Additional file 1: Table S1. Notation, input, training, prediction, and application of each model. Table S2. Imaging parameters for all sequences used in this study. Table S3. Mean, standard deviation and coefficient of variation (CV) of T1 of each phantom estimated by MOLLI and three MyoMapNet models. Table S4. Standard deviation and coefficient of variation (CV) of T1 of existing data estimated by MOLLI and MyoMapNet. Table S5. Standard deviation and coefficient of variation (CV) of T1 for prospectively collected data by MOLLI and LL4 with MyoMapNet. Table S6. Native, post-contrast T1, and corresponding ECV for existing MOLLI data estimated by curve-fitting methods. [file 12968_2021_834_MOESM1_ESM.docx]

**Appendix 1- Tables for parameters of MyoMapNet and imaging sequences, phantom results, and in-vivo T_1_ precision**

**Table S1.** Notation, input, training, prediction, and application of each model

| **MyoMapNet** | **Input**  **T_1_-weighted Signals** | **Training Dataset** | **Predication and Application** | **Evaluation** |
| --- | --- | --- | --- | --- |
| **4, PreGd** | 4 | MOLLI5(3)3 | Native T_1_, ECV with MyoMapNet ^4, PostGd^ | Existing MOLLI5(3)3 data,  prospectively phantom and native in-vivo data by LL4 |
| **4, PostGd** | 4 | MOLLI4(1)3(1)2 | Post-contrast T_1_ and ECV with MyoMapNet ^5, PreGd^ or MyoMapNet ^4, PreGd^ | Existing MOLLI5(3)3 data,  prospectively phantom and native in-vivo data by LL4 |
| **4, Pre+PostGd** | 4 | MOLLI5(3)3  and MOLLI4(1)3(1)2 | Native and post-contrast T_1_, ECV | Existing MOLLI5(3)3 data,  prospectively phantom and native in-vivo data by LL4 |
| **5, PreGd** | 5 | MOLLI5(3)3 | Native T_1_ , ECV with MyoMapNet ^4, PostGd^ | Only existing MOLLI5(3)3 data, |

**Table S2.** Imaging parameters for all sequences used in this study

| Parameter | IR-SE | CPMG-SE | MOLLI5(3)3 | MOLLI4(1)3(1)2 | LL4 |
| --- | --- | --- | --- | --- | --- |
| Readout Sequence | spin echo | spin echo | bSSFP | bSSFP | bSSFP |
| Field of View (R×P mm^3^) | 200×200 | 200×200 | 360×325 | 360×325 | 360×325 |
| Voxel Size (mm^3^) | 0.8×0.8×8 | 0.8×0.8×8 | 1.7×1.7×8 | 1.7×1.7×8 | 1.7×1.7×8 |
| Slice | 1 | 1 | 3 | 3 | 1 |
| Flip Angle (º) | 90 | 90 | 35 | 35 | 35 |
| TR | 10 s | 10 s | 2.5 ms | 2.5 ms | 2.5 ms |
| TE (ms) | 8 | 8.5 | 1.02 | 1.02 | 1.02 |
| Bandwidth (Hz/Pixel) | 250 | 250 | 1093 | 1093 | 1093 |
| GRAPPA Acceleration | - | - | R=2 | R=2 | R=2 |
| Partial Fourier | - | - | 7/8 | 7/8 | 7/8 |
| Lines of k-space per Shot | 1 | 1 | 75 | 75 | 75 |
| Acquisition Window  (ms) | - | - | 198 | 198 | 198 |
| T_1_- or T_2_- weighted Images | 14 | 8 | 8 | 9 | 4 |
| Inversion-Recovery/Echo Time (ms) | 100, 200, 300, 400, 500, 600, 700, 800, 900, 1000, 1500, 2000, 2500, 3000 | 8.5, 17.0, 25.5, 34.0, 42.5, 51.0, 59.5, 68.0 | 108,188 | 108,188,268 | 108 |

* IR-SE: Inversion-Recovery Spin-Echo; CPMG-SE: Carr-Purcell-Meiboom-Gill Spin-Echo; MOLLI: Modified Look-Locker inversion recovery. bSSFP: balanced Steady-State Free-Precision; R×P represents readout, phase; GRAPPA: GeneRalized Autocalibrating Partial Parallel Acquisition.

**Table S3.** Mean, standard deviation (SD) and coefficient of variation (CV) of T_1_ of each phantom estimated by MOLLI and three MyoMapNet models

|  | **IR-SE** | **CPMG-SE** | **MOLLI** | | | | **MyoMapNet** | | | | | |
| --- | --- | --- | --- | --- | --- | --- | --- | --- | --- | --- | --- | --- |
|  |  |  | **5(3)3** | | **4(1)3(1)2** | | **4, PreGd** | | **4, Pre+PostGd** | | **4, PostGd** | |
| **#** | **T_1_ (ms)** | **T_2_ (ms)** | **T_1_ (ms)** | **CV(%)** | **T_1_ (ms)** | **CV(%)** | **T_1_ (ms)** | **CV%** | **T_1_ (ms)** | **CV%** | **T_1_ (ms)** | **CV%** |
| **1** | 310 | 146 | 304±4 | 1.3±0.17 | 303±3.4 | 1.1±0.02 | 294±5.1 | 1.7±0.03 | 303±4.8 | 1.6±0.04 | 311±3.4 | 1.1±0.02 |
| **2** | 353 | 45 | 313±15 | 4.8±0.42 | 319±4.1 | 1.3±0.02 | 302±5.7 | 1.9±0.07 | 311±5.2 | 1.7±0.05 | 317±3.8 | 1.2±0.04 |
| **3** | 415 | 166 | 403±7 | 1.7±0.04 | 404±6.3 | 1.5±0.03 | 394±8.2 | 2.1±0.03 | 410±8.6 | 2.1±0.04 | 408±9.1 | 2.2±0.03 |
| **4** | 497 | 45 | 453±4 | 0.9±0.02 | 455±3.7 | 0.8±0.02 | 445±5.7 | 1.3±0.03 | 454±4.3 | 1.0±0.03 | 453±4.4 | 1.0±0.04 |
| **5** | 515 | 170 | 502±4.7 | 0.9±0.03 | 502±4.3 | 0.9±0.02 | 514±5.9 | 1.1±0.07 | 505±5.2 | 1.0±0.06 | 506±5 | 1.0±0.04 |
| **6** | 620 | 45 | 552±4.9 | 0.9±0.03 | 554±4.4 | 0.8±0.02 | 559±5.1 | 0.9±0.04 | 548±5.1 | 0.9±0.04 | 548±4.9 | 0.9±0.05 |
| **7** | 724 | 42 | 620±6.3 | 1.0±0.01 | 621±5.7 | 0.9±0.02 | 624±6.1 | 1.0±0.02 | 612±6.1 | 1.0±0.02 | 610±5.7 | 1.0±0.02 |
| **8** | 922 | 41 | 714±5.5 | 0.8±0.03 | - | - | 715±5.5 | 0.8±0.04 | 706±6.4 | 0.9±0.05 | - | - |
| **9** | 942 | 49 | 814±4.4 | 0.5±0.02 | - | - | 812±4.8 | 0.6±0.02 | 811±5.5 | 0.7±0.03 | - | - |
| **10** | 1177 | 43 | 986±6 | 0.6±0.02 | - | - | 982±6.6 | 0.7±0.05 | 981±7 | 0.7±0.06 | - | - |
| **11** | 1419 | 247 | 1367±10.3 | 0.8±0.03 | - | - | 1367±11.1 | 0.8±0.06 | 1365±12.4 | 0.9±0.06 | - | - |
| **12** | 1688 | 227 | 1563±12.9 | 0.8±0.02 | - | - | 1573±13.5 | 0.9±0.05 | 1573±13 | 0.9±0.05 | - | - |
| µ±σ | - | - | - | 1.3±1.2 | - | 1.0±0.3 | - | 1.2±0.5 | - | 1.1±0.4 | - | 1.2±0.5 |

*IR-SE: inversion-recovery spin-echo sequence; CPMG-SE: Carr-Purcell-Meiboom-Gill spin-echo; MOLLI: Modified Look-Locker inversion recovery;

** Vial #1-7 for post-contrast myocardium and blood; Vial #8-12 for pre-contrast myocardium and blood.

***T_1_, SD and CV are calculated by averaging the corresponding results of each measurement across all repetitions.

****All P>0.05 when compared to MOLLI5(3)3 or MOLLI4(1)3(1)2

**Table S4.** Standard deviation (SD) and coefficient of variation (CV) of T_1_ of existing data estimated by MOLLI and MyoMapNet

|  |  | **Myocardium** | | **Blood** | |
| --- | --- | --- | --- | --- | --- |
|  |  | **SD (ms)** | **CV (%)** | **SD (ms)** | **CV (%)** |
| **Native T_1_** | **MyoMapNet^4, Pre^** | 61.6±12.3 | 5.2±1.1 | 37.7±10.4 | 2.0±0.6 |
|  | **MyoMapNet^4, Pre+PostGd^** | 62.0±12.5 | 5.2±1.1 | 37.8±10.5 | 2.0±0.6 |
|  | **MyoMapNet^5, Pre^** | 61.4±12.2 | 5.2±1.1 | 36.8±10.2 | 2.0±0.5 |
|  | **MOLLI5(3)3** | 59.7±12.6 | 5.0±1.1 | 33.9±9.8 | 1.8±0.5 |
| **Post-Contrast T_1_** | **MyoMapNet^4, PostGd^** | 33.6±12.3 | 5.9±2.4 | 15.6±5.2 | 3.7±1.2 |
|  | **MyoMapNet^4, Pre+PostGd^** | 34.3±11.9 | 6.1±2.3 | 15.5±5.3 | 3.7±1.2 |
|  | **MOLLI4(1)3(1)2** | 29.4±9.5 | 5.2±1.8 | 9.2±2.5 | 2.2±0.6 |

*All P<0.05 when compared to MOLLI5(3)3 or MOLLI4(1)3(1)2

**Table S5.** Standard deviation (SD) and coefficient of variation (CV) of T_1_ for prospectively collected data by MOLLI and LL4

|  |  | **Myocardium** | | **Blood** | |
| --- | --- | --- | --- | --- | --- |
|  |  | **SD (ms)** | **CV (%)** | **SD (ms)** | **CV (%)** |
| **Native T_1_** | **MyoMapNet^4, Pre^** | 55.6±10.9 | 4.7±0.9 | 34.1±11.6^§^ | 1.9±0.7^§^ |
|  | **MyoMapNet^4, Pre+PostGd^** | 57.0±13.4 | 4.8±0.9 | 34.3±14.8^§^ | 1.9±0.8 |
|  | **MOLLI5(3)3** | 55.5±12.1 | 4.7±1.0 | 28.6±12.8 | 1.6±0.7 |
| **Post-Contrast T_1_** | **MyoMapNet^4, PostGd^** | 28.8±11.3 | 4.2±2.1 | 14.7±6.4^§^ | 2.7±1.4^§^ |
|  | **MyoMapNet^4, Pre+PostGd^** | 29.6±9.1^§^ | 4.3±1.8^§^ | 15.0±6.7^§^ | 2.7±1.4^§^ |
|  | **MOLLI4(1)3(1)2** | 26.1±9.1 | 3.8±1.4 | 9.4±7.9 | 1.7±1.4 |

^§^p-value < 0.05 when compared to MOLLI5(3)3 or MOLLI4(1)3(1)2
