# Supplementary material for: Accelerated cardiac T1 mapping in four heartbeats with inline MyoMapNet: a deep learning-based T1 estimation approach
Source: J Cardiovasc Magn Reson. 2022 Jan 6;24:6. doi: 10.1186/s12968-021-00834-0 (PMC8734349; doi:10.1186/s12968-021-00834-0)
Supplement: Supplementary file 2 — Additional file 2: Figure S1. Simulation results. Bland–Altman plots show the mean difference and 95% limits of agreement in simulated T1 with different signal-to-noise (SNR) between MyoMapNet and MOLLI5(3)3, and LL5-3P-fitting and MOLLI5(3)3. Figure S2. Representative in-vivo T1 and corresponding ECV maps from the different number of T1-weighted images of MOLLI using MyoMapNet and curve-fitting methods. Figure S3. Comparison between MyoMapNet and curve-fitting methods (LL4- and LL5-3P-fitting) for myocardium T1 from four or five T1 weighted images, using MOLLI as reference: MOLLI5(3)3 for native T1 and MOLLI4(1)3(1)2 for post-contrast T1. Each data point was averaged across three LV slices for each patient. Mean difference and 95% limits of agreement are indicated as red and dotted lines, respectively. Figure S4. Comparison of MyoMapNet and curve-fitting methods for blood T1 from a few T1-weighted images (4 or 5) to reference sequences. MOLLI5(3)3 was the reference for native T1, and MOLLI4(1)3(1)2 was the reference for post-contrast T1. Mean difference and 95% limits of agreement are indicated as red and dotted lines for each subfigure, respectively. Figure S5. Comparing ECV from MyoMapNet models and curve fitting (LL4- and LL5-3P-fitting) to MOLLI. Mean difference and 95% limits of agreement are indicated as red and dotted lines for each subfigure, respectively. Native and post-contrast T1 values used for ECV measurement are indicated for each method. [file 12968_2021_834_MOESM2_ESM.docx]

**Appendix 2- Comparison between classical curve fitting and deep learning for Look-Locker with four or five T_1_ weighted images**

We performed numerical simulations and in-vivo studies of MOLLI5(3)3 to compare MyoMapNet performance versus the classical curve fitting method for estimating T_1_ from a reduced number MOLLI5(3)3 T_1_ weighted images.

MOLLI5(3)3 simulations were performed to generate testing signal-intensity time courses. A single-shot readout was simulated using Bloch equation with the following parameters: balanced Steady-State Free Precession (bSSFP) acquisition with five ramp-up pulses, TR/flip angle =2.5 ms/35º, 80 phase-encode lines, acquisition window = 200 ms, and linear phase ordering. The time between inversion and k-space center was 120 ms and 200 ms for two LL experiments of MOLLI5(3)3. Simulated T_1_ ranged from 400 ms to 1800 ms at an increment of 0.1 ms, T_2_ was 42 ms, and heart rate was 60 bpm. Gaussian noise was added to the simulated signals to achieve different SNRs (20, 40, and 100). The simulated testing dataset contained 16,000 samples. A 3-parameter fitting method with Look-Locker correction and MyoMapNet^5, PreGd^ were used to calculate T_1_ for each signal time course.

Furthermore, we used the curve-fitting method to estimate T_1_ from four or five T_1_ weighted images of MOLLI in the existing MOLLI dataset. For the native T_1_ data, we fitted T_1_ using four and five T_1_-weighted images. For post-contrast data, we only used four T_1_-weighted images. We named these curve fitting studies LL4-3^P^-fitting and LL5-3^P^-fitting, respectively.

Bland-Altman plots show differences in T_1_ between MyoMapNet and MOLLI5(3)3 for simulations of different noise levels, as well as a comparison between LL5-3^P^-fitting and MOLLI5(3)3 (**Figure S1**)**.** For higher SNR, T_1_ values from LL5-3^P^-fitting exhibit systematic error as a function of the T_1_ value. For the same SNR, MyoMapNet shows no systematic error for different T_1_ values. As SNR decreases, there is an increase in estimated T_1_ errors using both techniques, but with lower error in MyoMapNet (bias: 0±15 ms; 95% CI: from -0.3 to 0.1) compared to LL5 (bias: -3±32 ms; 95% CI: from -4 to -3; P <0.001) relative to MOLLI5(3)3 at SNR=40. At SNR of 20, the mean difference between MyoMapNet and MOLLI5(3)3 (bias: 0±31ms; 95% CI: from -1 to 1) is smaller than that between LL5-3^P^-fitting and MOLLI5(3)3 (bias: -3±54 ms; 95% CI: from -4 to -3; P <0.001).

Visually, T_1_ and ECV maps measured from either four or five MOLLI T_1_-weighted images using either MyoMapNet or the classical curve fitting have comparable image quality to MOLLI (**Figure S2)**. The mean absolute difference in native and post-contrast T_1_ between MyoMapNet and MOLLI was smaller than that between curve-fitting and MOLLI (**Supporting Figures S3 and S4**). The 95% CI of T_1_ difference between MyoMapNet and MOLLI was also smaller than those between LL4/ LL5-3^P^-fitting methods and MOLLI. For curve fitting, the mean difference in ECV was ~0.6% with 95% CI from -2.2% to 3.5%, which was larger than that between MyoMapNet and MOLLI (bias: 0.40; 95%CI: from -1% to 2%).

In **Supporting Table S6**, standard deviation for native myocardium and blood T_1_ from LL4/ LL5-3^p^-fitting were higher than those from MyoMapNet and MOLLI5(3)3. Standard deviation for post-contrast myocardium and blood T_1_ from LL4/ LL5-3^p^-fitting were also higher than those from MyoMapNet and MOLLI5(3)3. Both native and post-contrast T_1_ from LL4-3^p^-fitting or LL5-3^p^-fitting had the highest coefficient of variation (CV) values compared to MOLLI and MyoMapNet: 5.8% and 6.6% for myocardium; 2.5% and 2.1% for native blood; and 4.5% for post-contrast blood.

In summary, for the cardiac T_1_ mapping with a reduced number of T_1_-weighted images compared to the original MOLLI, MyoMapNet had higher T_1_ accuracy and precision than the conventional fitting method.

**
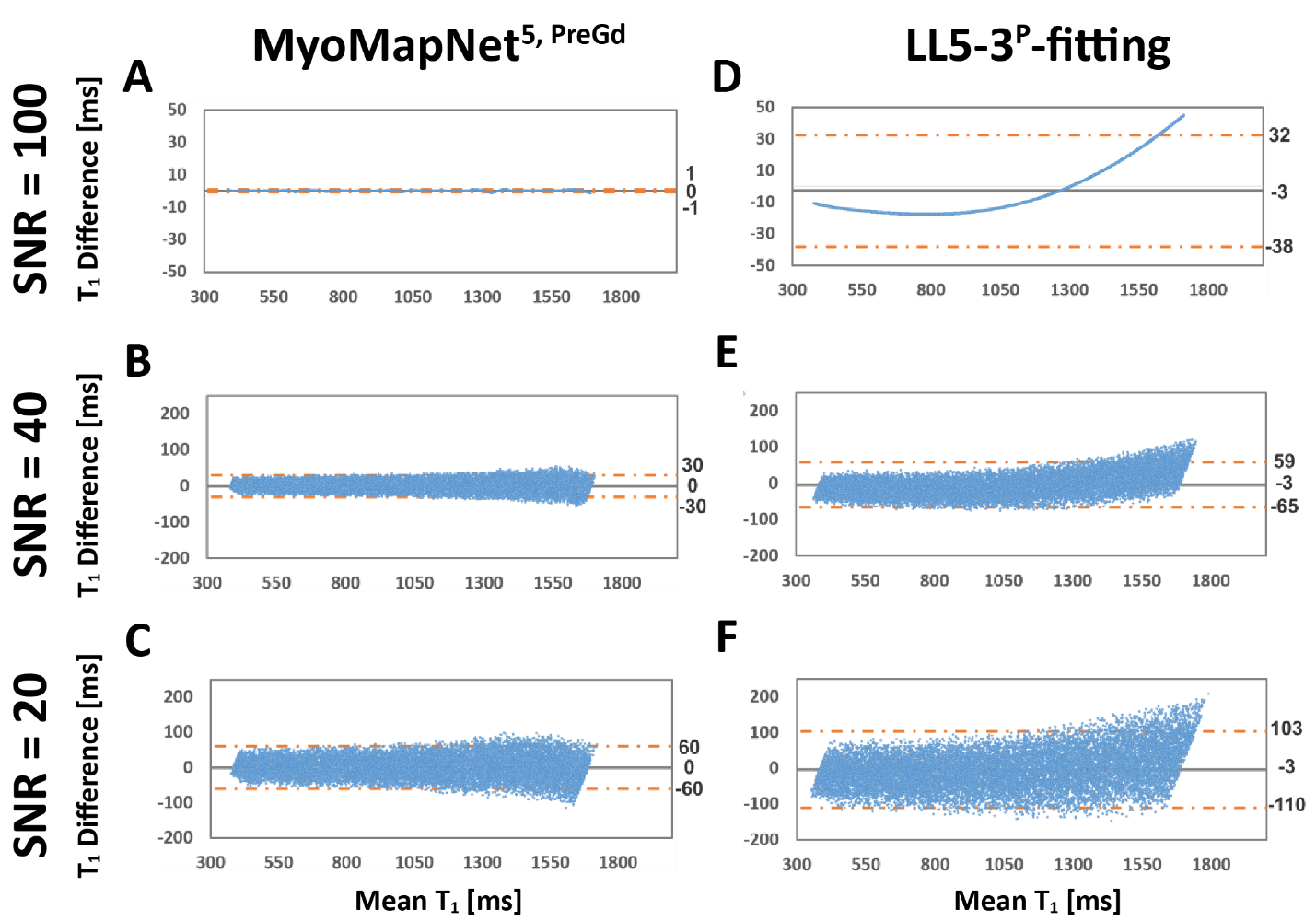
**

**Supporting Figure S1:** Simulation results. Bland-Altman plots show the mean difference and 95% limits of agreement in simulated T_1_ with different SNR between MyoMapNet and MOLLI5(3)3, and LL5-3^P^-fitting and MOLLI5(3)3.


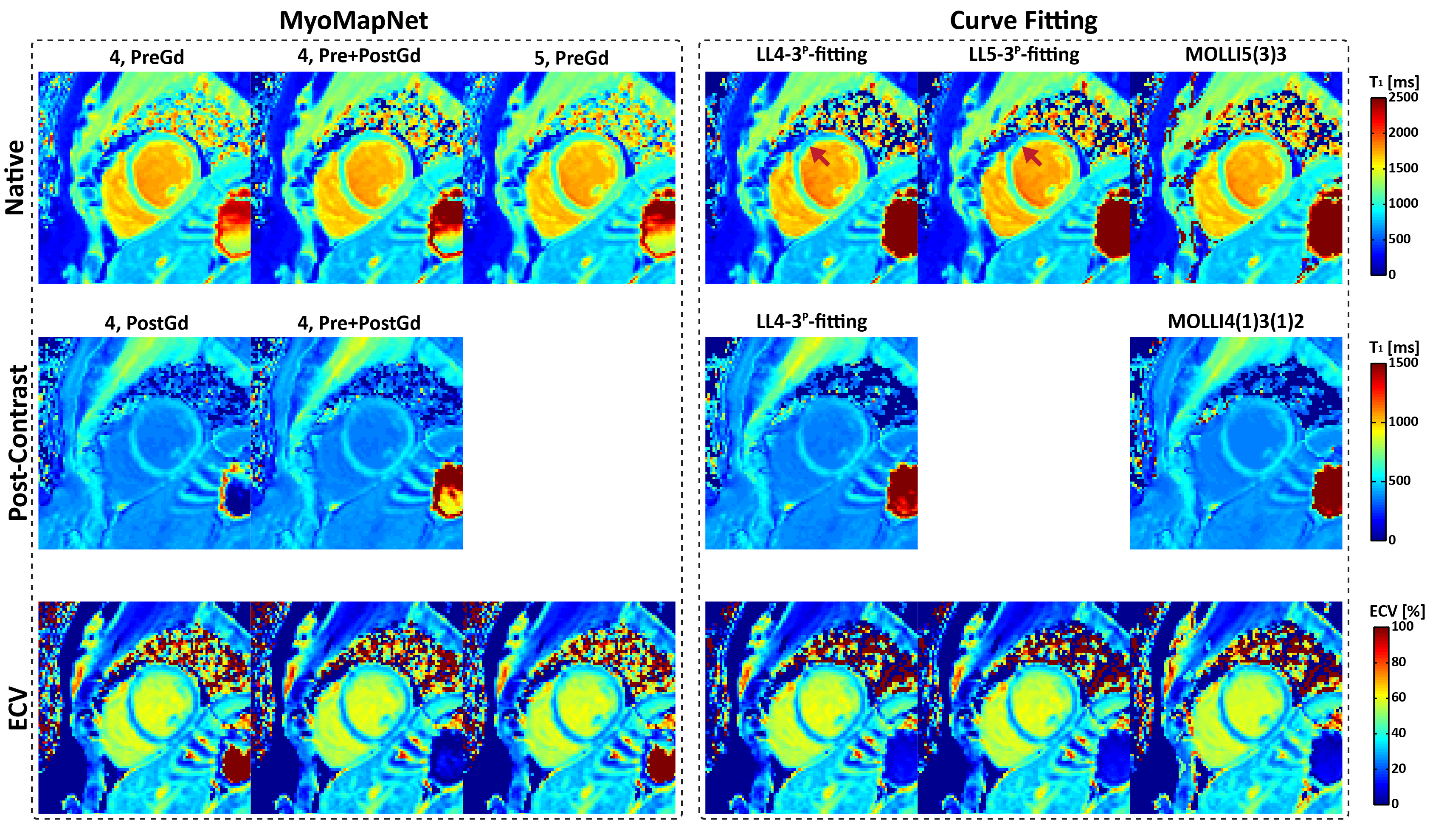


**Supporting Figure S2.** Representative in-vivo T_1_ and corresponding ECV maps from the different number of T_1_-weighted images of MOLLI using MyoMapNet and curve-fitting methods.


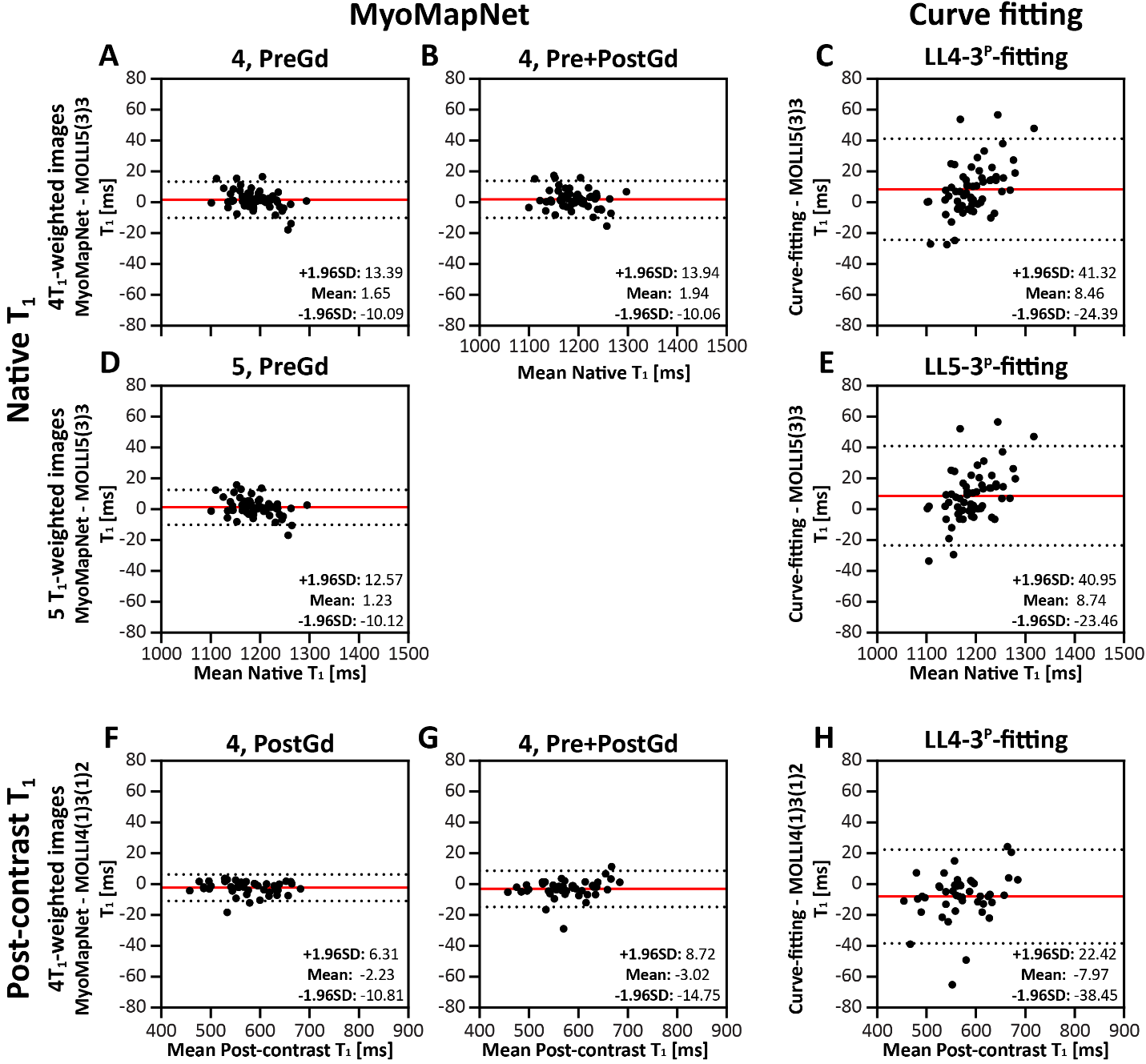


**Supporting Figure S3:** Comparison between MyoMapNet and curve-fitting methods (LL4- and LL5-3P-fitting) for myocardium T_1_ from four or five T_1_ weighted images, using MOLLI as reference: MOLLI5(3)3 for native T_1_ and MOLLI4(1)3(1)2 for post-contrast T_1_. Each data point was averaged across three left-ventricular slices for each patient. Mean difference and 95% limits of agreement are indicated as red and dotted lines, respectively.


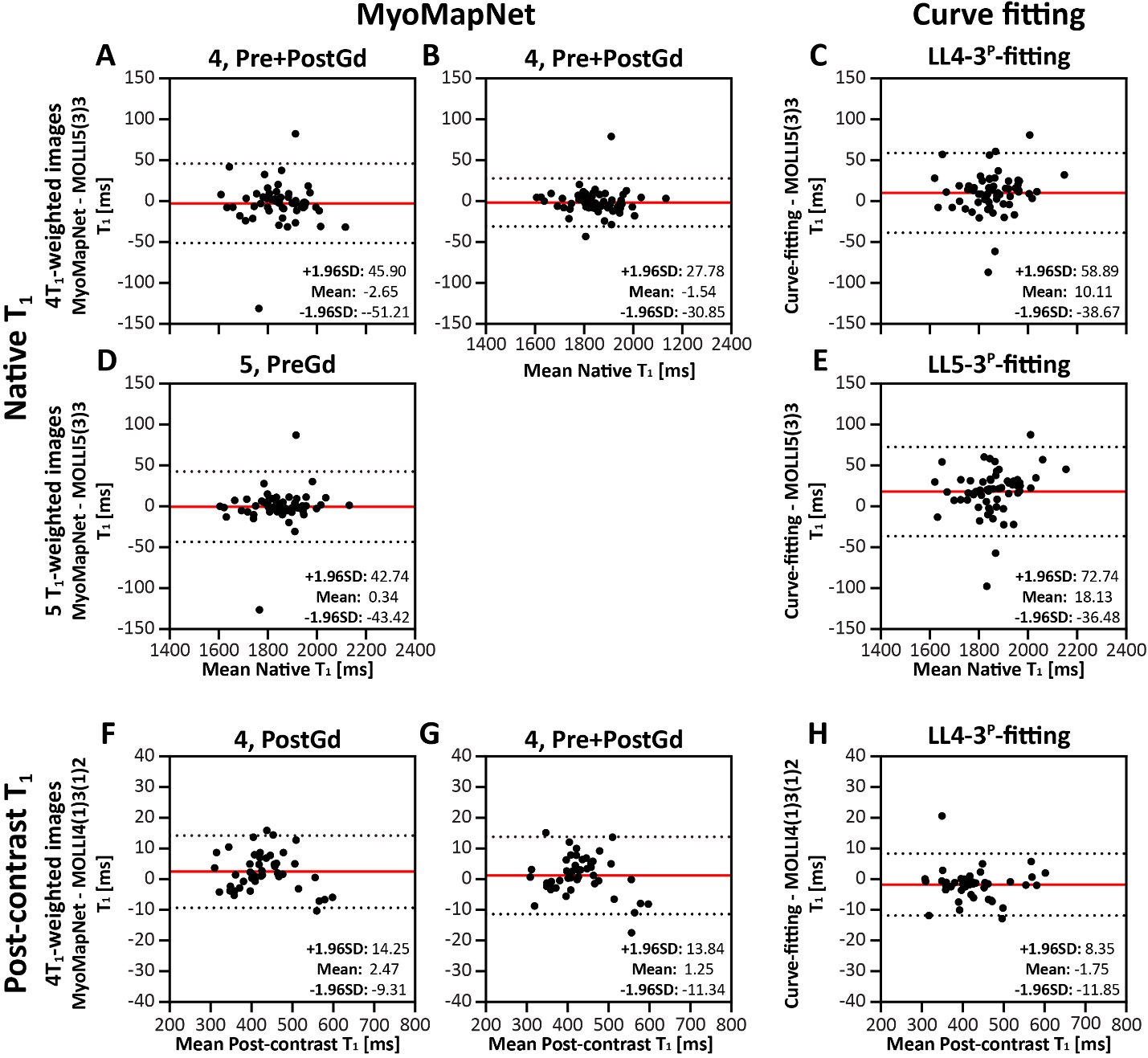


**Supporting Figure S4:** Comparison of MyoMapNet and curve-fitting methods for blood T_1_ from a few T_1_-weighted images (4 or 5) to reference sequences. MOLLI5(3)3 was the reference for native T_1_, and MOLLI4(1)3(1)2 was the reference for post-contrast T_1_. Mean difference and 95% limits of agreement are indicated as red and dotted lines for each subfigure, respectively.


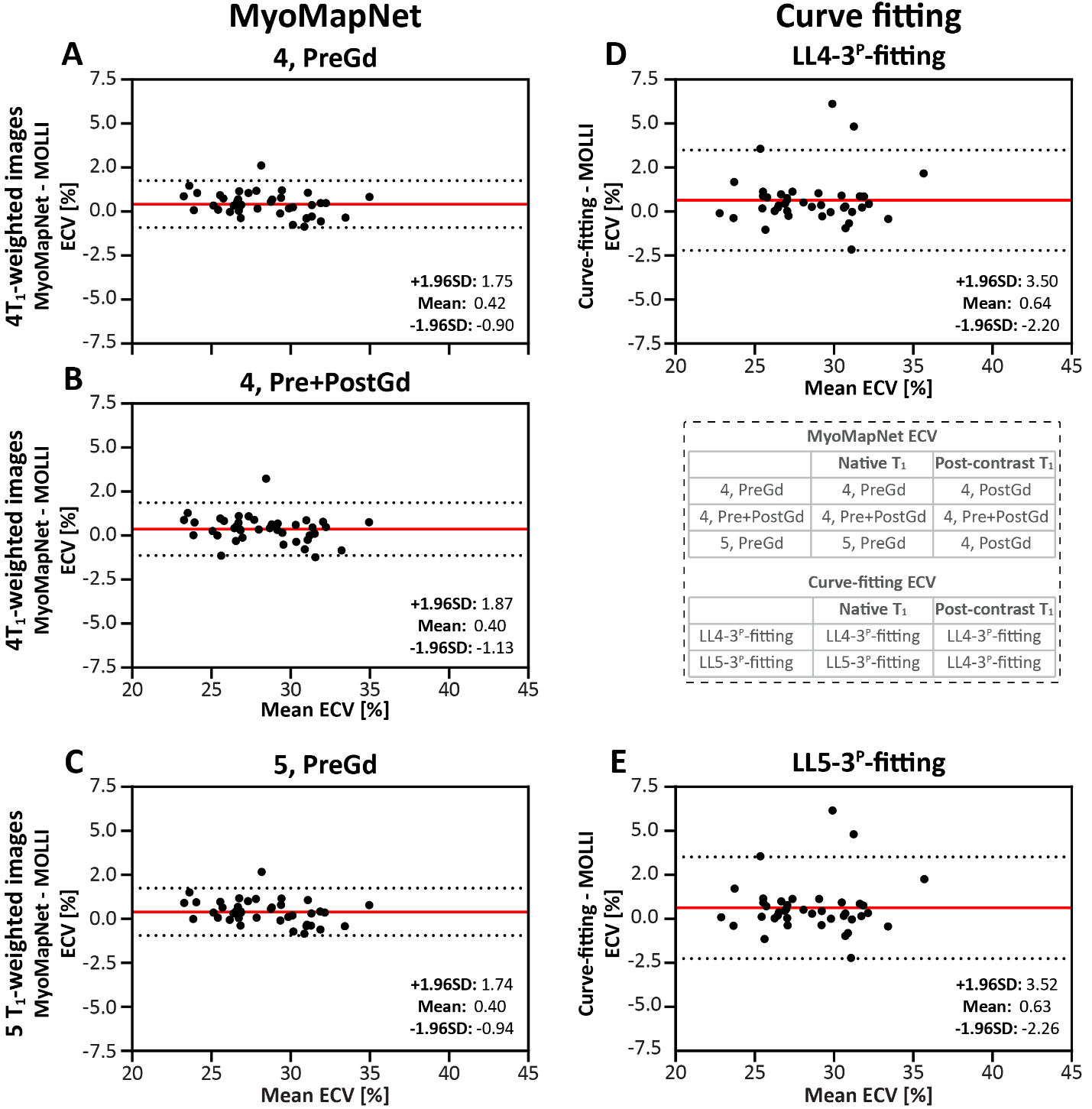
**Supporting Figure S5.** Comparing ECV from MyoMapNet models and curve fitting (LL4- and LL5-3^P^-fitting) to MOLLI. Mean difference and 95% limits of agreement are indicated as red and dotted lines for each subfigure, respectively. Native and post-contrast T_1_ values used for ECV measurement are indicated for each method.

**Supporting Table S6.** Native, post-contrast T_1_, and corresponding ECV for existing MOLLI data estimated by curve-fitting methods.

|  |  | | **Myocardium** | **Blood** |
| --- | --- | --- | --- | --- |
| **Native T_1_** | **LL4-3^P^-fitting** | Mean (ms) | 1197±49 | 1860±103 |
|  |  | SD (ms) | 69.6±15.9 | 47.0±15.0 |
|  |  | CV (%) | 5.8±1.5 | 2.5±0.8 |
|  | **LL5-3^P^-fitting** | Mean (ms) | 1197±49 | 1868±106 |
|  |  | SD (ms) | 69.7±16.0 | 39.5±11.4 |
|  |  | CV (%) | 5.8±1.5 | 2.1±0.5 |
| **Post-Contrast T_1_** | **LL4-3^P^-fitting** | Mean (ms) | 568±59 | 428±70 |
|  |  | SD (ms) | 37.0±11.2 | 18.6±8.0 |
|  |  | CV (%) | 6.6±2.2 | 4.5±2.0 |
| **ECV (%)** | **LL4-3^P^-fitting** | | 28.8±3.0 | |
|  | **LL5-3^P^-fitting** | | 28.8±3.0 | |

*ECV: Extracellular volume; SD: standard deviation; CV: Coefficient of variation

**Mean, SD, and CV of T_1_ were calculated by averaging the corresponding results of each subject across all subjects
